# Hospital-Acquired Pressure Ulcers and Long-Term Motor Score Recovery in Patients With Acute Cervical Spinal Cord Injury

**DOI:** 10.1001/jamanetworkopen.2024.44983

**Published:** 2024-12-06

**Authors:** Marcel A. Kopp, Felix W. Finkenstaedt, Oliver Schweizerhof, Ulrike Grittner, Peter Martus, Ralf Watzlawick, David Brienza, Vieri Failli, Yuying Chen, Michael J. DeVivo, Jan M. Schwab

**Affiliations:** 1 Department of Neurology and Experimental Neurology, Clinical and Experimental Spinal Cord Injury Research (Neuroparaplegiology), Charité - Universitätsmedizin Berlin, corporate member of Freie Universität Berlin, Humboldt-Universität zu Berlin, and Berlin Institute of Health, Berlin, Germany; 2 Berlin Institute of Health, QUEST—Center for Transforming Biomedical Research, Berlin, Germany; 3 Institute of Biometry and Clinical Epidemiology, Charité–Universitätsmedizin Berlin, corporate member of Freie Universität Berlin, Humboldt-Universität zu Berlin, and Berlin Institute of Health, Berlin, Germany; 4 Berlin Institute of Health, Berlin, Germany; 5 Department of Clinical Epidemiology and Applied Biostatistics, Eberhard Karls Universität Tübingen, Tübingen, Germany; 6 Department of Neurosurgery, Freiburg University Medical Center, Freiburg, Germany; 7 Department of Rehabilitation Science and Technology, University of Pittsburgh, Pennsylvania; 8 National Spinal Cord Injury Statistical Center, Department of Physical Medicine and Rehabilitation, University of Alabama at Birmingham; 9 Department of Neurology, Spinal Cord Division, The Neurological Institute, The Ohio State University, Wexner Medical Center, Columbus; 10 Department of Neuroscience and Physical Medicine and Rehabilitation, The Neurological Institute, The Ohio State University, Wexner Medical Center, Columbus; 11 Belford Center for Spinal Cord Injury, The Ohio State University, Wexner Medical Center, Columbus

## Abstract

**Importance:**

Pressure ulcers (PUs) are (1) prevalent secondary complications after spinal cord injury (SCI), (2) present with elevated systemic inflammatory tone, and (3) may interfere with healing processes underlying neurological recovery (disrepair).

**Objective:**

To investigate whether PUs acquired during initial hospitalization are associated with neurological and functional long-term outcome and survival after SCI.

**Design, Setting, and Participants:**

Multicenter cohort study at 20 centers of the prospective SCI Model Systems (SCIMS) Database (Birmingham, AL). Patients with acute traumatic cervical SCI with relevant motor impairment (ie, American Spinal Injury Association [ASIA] impairment scale [AIS] A, B, and C) were enrolled from January 1996 to September 2006 and followed up until June 2016. Data were analyzed from April 2021 to September 2024.

**Exposures:**

PUs acquired during surgical or first rehabilitative SCI care.

**Main outcomes and measures:**

The change in the ASIA motor score at 1 year after SCI was the primary end point. Secondary end points included the recovery of functional independence measure (FIM) motor score at 1 year after SCI and mortality up to 10 years. ASIA and FIM motor score were analyzed applying linear mixed models with random intercept adjusted for baseline neurological level, AIS, and sociodemographic factors. Mortality was analyzed using Cox regression.

**Results:**

The study included 1282 patients with a mean (SD) age of 38.0 (15.7) years and consisted of 1028 (80.2%) male patients. Regarding race and ethnicity, 349 of 1249 (27.9%) were African American patients, 1139 of 1273 (89.5%) were non-Hispanic patients, and 834 of 1249 (66.8%) were White patients. During initial hospitalization, 594 patients (45.7%) acquired PUs. Exposure to PUs was associated with impaired motor recovery 1 year after SCI compared with unexposed patients (−9.1 ASIA motor score points; 95% CI, −12.3 to −6.0; *P* < .001). In addition, PUs were associated with lower recovery of physical independence 1 year after SCI (−8.3 FIM motor score points; 95% CI: −11.1 to −5.5; *P* < .001). Cox regression confirmed PUs as a risk marker for death up to 10 years after SCI (hazard ratio, 1.41; 95% CI, 1.09 to 1.82; *P* = .01).

**Conclusions and Relevance:**

In this cohort study, PUs acquired during initial hospitalization after SCI were independently associated with poor long-term neurofunctional outcome. PUs constitute a modifiable factor associated with risk for worse long-term disability (recovery confounder) and elevated mortality.

## Key Points

**Question:**

Are hospital-acquired pressure ulcers after acute spinal cord injury associated with worse neurological outcome?

**Findings:**

This cohort study included 1282 individuals with spinal cord injury, of which 594 (45.7%) developed pressure ulcers during initial hospitalization. Patients with pressure ulcers regained significantly less motor function up to 1 year after injury (−9.1 total American Spinal Injury Association motor score points) and their recovery of independence in activities of daily living was significantly restricted (−8.3 functional independence measure motor score points) compared with unexposed patients.

**Meaning:**

These findings suggest pressure ulcers represent a preventable outcome-modifying factor after acute traumatic spinal cord injury.

## Teaser

This cohort study evaluates whether pressure ulcers acquired during initial hospitalization are associated with neurological and functional long-term outcome and survival among patients with spinal cord injuries.

## Introduction

Considerable outcome variability after spinal cord injury (SCI) is not explained by injury severity alone and implies the presence of secondary outcome modifying factors that disturb lesion-affected and recovery-related networks.^1^ Pressure ulcers (PUs) are a prevalent comorbidity acquired after SCI and increase systemic inflammatory levels to allow for signaling far beyond their localized appearance. Thus, PUs may interfere with repair mechanisms (disrepair) affecting the entire body to also undermine endogenous neurological recovery potential after SCI.^2,3^

Acquired PUs constitute a supernumeric inflammatory lesion in addition to the SCI itself. PUs are associated with bacteremia and can convert into osteomyelitis.^4,5,6^ The skewed PU-associated inflammatory tone is mirrored by elevated, nonresolving, systemic C-reactive protein,^7,8^ interleukin-2 receptor,^9^ or interferon gamma-induced protein 10/C-X-C motif chemokine ligand levels^10^ as a candidate mechanism to interfere with homeostatic wound healing processes.^11^ A nonresolving systemic inflammatory response can signal across the blood-brain and/or –spinal cord barrier and cause neuroaxonal injury,^12^ neurodegeneration,^13,14^ and thus restrict the neuronal plasticity reservoir.^15^ Of note, patients with SCI and acquired PUs also demonstrate a hypermetabolic and/or dysmetabolic state signifying additional wide and systemic alterations.^16,17^

It remains elusive whether PUs as supernumeric inflammatory lesions are associated with outcome alteration of neurological diseases (phenotype).^18^ PUs are among the most prevalent SCI-associated comorbidities and may result in disrepair.^19^ We analyzed the differential association of hospital-acquired (nosocomial) PUs with neurological and functional long-term outcomes as well as survival after acute traumatic cervical SCI by interrogation of the largest worldwide prospective multicenter SCI database.

## Methods

### Study Design

The cohort study was conducted in datasets from 20 SCI centers contributing to the prospective multicenter SCI Model Systems (SCIMS) Database.^20,21^ Patients with at least 1 acquired PU during initial hospitalization (hereafter referred to as the exposed group) were compared with patients without (hereafter referred to as the unexposed group). The review boards of each SCIMS center approved the database, and enrollment was in accordance with the Declaration of Helsinki. All participants were informed about the aim of the study and gave their written informed consent. The study is reported according to the Strengthening the Reporting of Observational Studies in Epidemiology (STROBE) reporting guideline.^22^

### Setting

The SCIMS are specialized SCI care centers (trauma centers and inpatient rehabilitation centers) aiming to implement standards of medical SCI care and research and to disseminate knowledge about SCI. The SCIMS database, designed for epidemiological and health services research in SCI,^20,21^ collects health care and outcome data of patients with acute traumatic SCI during the initial hospital care and also at follow-up at postinjury year 1, year 5, and then every 5 years thereafter.

### Population

Patients with traumatic SCI admitted within 24 hours after injury to a SCIMS center and enrolled from January 1996 to September 2006 were screened because during this project period uniform definitions of the number and grade of PUs acquired during hospitalization were applied. Cervical neurological level of injury (NLI) and substantial motor impairment as defined by the American Spinal Cord Injury Association (ASIA) impairment scale (AIS) grade A, B, or C (eTable 1 in Supplement 1) were chosen as inclusion criteria to prevent higher heterogeneity related to thoracic or lumbar NLI or to avoid ceiling effects in the outcome measures applicable for AIS D patients, respectively.^23^ Exclusion criteria were being aged less than 16 or more than 75 years or a stay in nursing home before injury. Additionally, patients were excluded if neurological baseline examinations were missing or not reasonable due to medical conditions such as concomitant injuries affecting consciousness. Furthermore, patients without information on the number or grade of PUs during initial hospitalization were excluded (eFigure 1 in Supplement 1). Self-reported race and ethnicity (African American, Asian or Pacific Islander, Hispanic, non-Hispanic, White, and Other) were assessed to consider possible associations of these variables with exposure and/or outcome.

### Data Collection

Procedures of data collections in the SCIMS and assurance of data quality and validity for this project are described in the data dictionaries (2000-2005 and 2006-2011).^24^ In this study, information on PUs acquired during acute surgical care and/or inpatient rehabilitation (ie, between admission to a study center within 24 hours after SCI and discharge from subsequent inpatient rehabilitation) were used as exposure variables. The PU variable was dichotomized; that is, patients with at least 1 PU of severity grade 1 to 4 (eTable 2 in Supplement 1) were handled as the exposed group and compared with the group of unexposed patients. For further information on variable definitions, see eMethods in Supplement 1.

### Outcomes

Neurological and functional outcome data were collected (1) at admission to acute surgical care and/or inpatient rehabilitation (baseline), (2) at discharge from inpatient rehabilitation (discharge), and (3) at the first annual examination after SCI (1 year) (eFigure 1 in Supplement 1). The International Standards for Neurological Classification of Spinal Cord Injury^25^ were applied for neurological assessments. The primary outcome was the change in total ASIA motor score (eTable 1 in Supplement 1). Secondary outcomes are changes in the upper extremity motor score (UEMS) and lower extremity motor score (LEMS) subscales, AIS (eTable 1 in Supplement 1), functional independence measure (FIM) motor items (eTable 3 in Supplement 1), and survival up to 10 years after SCI.

### Statistical Analysis

To compare the primary outcome, ASIA motor score, and the secondary outcomes, UEMS, LEMS, and FIM motor score, within and between the exposed and unexposed groups over time, linear mixed regression models with random intercepts (for centers and individuals nested in centers) were used with restricted maximum likelihood as the estimation method. The models were baseline adjusted and included time × PU group interaction. Subsequently, the models were adjusted for age, sex, educational status, native language status, occupational status, and NLI at baseline. Additional adjustment for baseline AIS was done in a separate model because AIS is a major prognostic factor for neurological and functional recovery.^23^ The estimated difference in marginal means between the exposed and unexposed groups was calculated for each follow-up time point (discharge from inpatient rehabilitation and 1 year) to describe the associations of PUs with neurological and functional outcome. The secondary end point, PU-associated mortality up to 10 years after SCI, was analyzed using the Kaplan-Meier method. Cox proportional hazards models with a random intercept for each center (frailty model) were used to estimate relative mortality risk and were adjusted for age, sex, occupational status, AIS, and NLI at baseline. All models were calculated in the total sample or stratified for the AIS grades (A, B, C) at baseline to examine associations within SCI severity subgroups. Further secondary outcome analyses (ie, change in the AIS from baseline to 1 year [AIS-conversion] or unadjusted change in ASIA motor score, UEMS, LEMS, and FIM motor score) as well as post hoc analyses of associations of combined exposures to PUs and infections or associations of the period of PU onset with changes in ASIA motor score, UEMS, LEMS, and FIM motor score were performed in an explorative manner and primarily with descriptive methods.

Sensitivity analyses to explore the dataset for evidence of possible selection or attrition bias comprised descriptive comparison of baseline characteristics of included vs excluded cases (eTable 4 in Supplement 1) or of available cases vs cases lost to follow-up at 1 year, respectively (eTable 5 in Supplement 1). In addition, the linear mixed model of the primary outcome, ASIA motor score, was recalculated after multiple imputation of missing values (see eMethods in Supplement 1). All statistical tests were 2-sided and the level of significance was .05.

For mixed regression models, the lmer function of the package lme4^26^ and for multiple imputation the mice package^27^ were used in R version 4.2.2 (R Project for Statistical Computing). All other analyses were performed using SPSS Statistics for Windows version 27.0.1 (IBM Corp). All bar graphs and violin plots were drawn using GraphPad Prism version 8.3 (GraphPad Software Inc). Considerations for regression model development were plotted using directed acyclic graphs (eFigure 2 in Supplement 1) with the browser-based application DAGitty version 3.1 (DAGitty).^28^

## Results

### Study Population

Of 3654 datasets screened, 1282 met the eligibility criteria and were analyzed. The process of data selection and numbers of excluded patients for each criterion are reported in eFigure 1 in Supplement 1. The study population had a mean (SD) age of 38.0 (15.7) years and included 1028 (80.2%) male patients. Regarding race and ethnicity, 349 of 1249 (27.9%) were African American patients, 1139 of 1273 (89.5%) were non-Hispanic patients, and 834 of 1249 (66.8%) were White patients. The grade of SCI severity was AIS A in 704 (54.9%), AIS B in 261 (20.4%), and AIS C in 317 (24.7%) individuals.

At least 1 PU occurred in 586 patients (45.7%) during acute care and/or inpatient rehabilitation. Stratified for SCI severity at baseline (AIS), 389 of 704 patients (55.3%) classified as AIS A, 112 of 261 (42.9%) classified as AIS B, and 85 of 317 (26.8%) classified as AIS C developed PUs. Patients with acquired PUs were assigned to the exposed group and compared with the unexposed group. The times of data collection for the entire study population and the exposed and unexposed groups are reported in eFigure 1 in Supplement 1.

### Baseline Characteristics of Exposed and Unexposed Patients

The exposed group included slightly more younger, male, and White patients, and fewer patients of Hispanic origin compared with the unexposed group. No substantial differences between the groups were observed in education level, English language status, occupational status, and marital status. The exposed group had more traffic accidents as traumatic cause, higher rates of complete SCI (AIS A) and upper cervical NLI (C1-C4), and lower ASIA motor score and FIM motor score values at baseline (Table 1).

**Table 1.** Sociodemographic and Clinical Patient Characteristics^a^

Characteristic	Patients, No. (%)		*P* value
	Unexposed to PUs (n=696)^b^	Exposed to PUs (n=586)^c^	
Age, mean (SD), y	38.9 (15.7)	36.9 (15.6)	.02
Sex			
Male	536 (77.0)	492 (84.0)	.002
Female	160 (23.0)	94 (16.0)	
Race			
African American	182 (27.0)	167 (29.1)	.01
Asian or Pacific Islander	18 (2.7)	8 (1.4)	
White	444 (65.3)	390 (67.9)	
Other^d^	31 (4.6)	9 (1.6)	
Hispanic origin			
Hispanic	84 (12.2)	50 (8.6)	.04
Non-Hispanic	607 (87.8)	532 (91.4)	
Language			
Primary language English	502 (93.3)	418 (94.4)	.34
Speaks/understands English	14 (2.6)	14 (3.2)	
Does not speak/understand English	22 (4.1)	11 (2.5)	
Education			
No high school	176 (29.7)	132 (26.8)	.58
High school	357 (60.2)	309 (62.8)	
College/university	60 (10.1)	51 (10.4)	
Occupational status			
Working/studying	438 (70.1)	362 (71.8)	.52
Unemployed/retired	187 (29.9)	142 (28.2)	
Marital status			
Married	250 (38.2)	178 (33.8)	.12
Unmarried	405 (61.8)	348 (66.2)	
Traumatic cause			
Traffic	316 (45.5)	315 (53.8)	.002
Falls	166 (23.9)	128 (21.8)	
Violence	85 (12.2)	64 (10.9)	
Sports	93 (13.4)	70 (11.9)	
Other	35 (5.0)	9 (1.5)	
AIS			
AIS A	315 (45.3)	389 (66.4)	<.001
AIS B	149 (21.4)	112 (19.1)	
AIS C	232 (33.3)	85 (14.5)	
NLI			
C1-C4	336 (49.2)	318 (54.9)	.04
C5-C8	347 (50.8)	261 (45.1)	
ASIA motor score points, median (IQR)	19.5 (6.0-38.0)	11.0 (4.0-23.0)	<.001
UEMS score points, median (IQR)	14.0 (5.3-25.0)	10.0 (2.5-20.0)	<.001
LEMS score points, median (IQR)	0.0 (0.0-11.0)	0.0 (0.0-0.0)	<.001
FIM motor score points, median (IQR)	14.0 (13.0-21.0)	13.0 (13.0-15.0)	<.001
FIM self-care subscore points, median (IQR)	6.0 (6.0-12.0)	6.0 (6.0-7.0)	<.001
FIM sphincter control subscore points, median (IQR)	2.0 (2.0-2.0)	2.0 (2.0-2.0)	.01
FIM transfer subscore points, median (IQR)	3.0 (3.0-3.0)	3.0 (3.0-3.0)	<.001
FIM mobility subscore points, median (IQR)	2.0 (2.0-2.0)	2.0 (2.0-2.0)	<.001

Abbreviations: ASIA, American Spinal Injury Association; AIS, ASIA impairment scale; FIM, Functional Independence Measure; LEMS, lower extremity motor score; NLI, neurological level of injury; PU, pressure ulcer; UEMS, upper extremity motor score.

^a^ Sociodemographic characteristics at the time of spinal cord injury and the neurological baseline comparing unexposed with exposed patients. The *t* test was used for age and the Mann-Whitney test for ASIA motor score, UEMS, LEMS, FIM motor score, and FIM subscores; the χ^2^ test was used for categorical variables.

^b^ Data were missing for the following variables and numbers of patients in the unexposed group: 21 missing race, 5 missing Hispanic origin, 158 missing language, 103 missing education, 71 missing occupational status, 41 missing marital status, 1 missing traumatic cause, 13 missing neurological level, 16 missing ASIA motor score, 12 missing UEMS, 6 missing LEMS, 69 missing FIM motor score, 65 missing FIM self-care subscore, 65 missing FIM sphincter control subscore, 66 missing FIM transfer subscore, and 67 missing FIM mobility subscore.

^c^ Data were missing for the following variables and numbers of patients in the exposed group: 12 missing race, 4 missing Hispanic origin, 143 missing language, 94 missing education, 82 missing occupational status, 60 missing marital status, 7 missing neurological level, 12 missing ASIA motor score, 9 missing UEMS, 6 missing LEMS, 20 missing FIM motor score, 19 missing FIM self-care subscore, 19 missing FIM sphincter control subscore, and 17 missing FIM transfer subscore, 17 missing FIM mobility subscore.

^d^ Includes respondents who selected Native American, Eskimo, or Aleut and other/unclassified.

### PU Onset, Number per Patient, and Severity

Within the exposed group, PUs occurred in 373 of 586 patients (63.7%) during acute care and in 213 patients (36.3%) during subsequent inpatient rehabilitation (Table 2). Regarding the number of PUs per patient, 347 patients (59.2%) had 1 PU, 141 (24.1%) had 2 PUs, and 98 (16.7%) had more than 2 PUs. The most severe PU was staged grade 1 in 126 patients (21.8%), grade 2 in 300 patients (51.8%), grade 3 in 93 patients (16.1%), and grade 4 in 60 patients (10.4%). Minor differences were observed between the AIS grades regarding number, maximum severity, and onset of PUs (Table 2).

**Table 2.** Characteristics of Hospital-Acquired Pressure Ulcers (PUs)

Characteristic	Participants, No. (%)^a^			
	Any AIS category (N = 586)	AIS A (n = 389)	AIS B (n = 112)	AIS C (n = 85)
No. of PUs				
1	347 (59.2)	219 (56.3)	71 (63.4)	57 (67.1)
2	141 (24.1)	105 (27.0)	20 (17.9)	16 (18.1)
>2	98 (16.7)	65 (16.7)	21 (18.8)	12 (14.1)
Grade of worst PU^b^				
1	126 (21.8)	78 (20.3)	25 (22.5)	23 (27.4)
2	300 (51.8)	196 (51.0)	57 (51.4)	47 (56.0)
3	93 (16.1)	67 (17.4)	17 (15.3)	9 (10.7)
4	60 (10.4)	43 (11.2)	12 (10.8)	5 (6.0)
PU onset				
Acute care	373 (63.7)	241 (62.0)	74 (66.1)	58 (68.2)
Inpatient rehabilitation	213 (36.3)	148 (38.0)	38 (33.9)	27 (31.8)

Abbreviations: ASIA, American Spinal Injury Association; AIS, ASIA impairment scale.

^a^ Data on grade of worst PU were missing for the following categories and numbers of patients: 5 missing for AIS A, 1 missing for AIS B, and 1 missing for AIS C.

^b^ PU grade of severity: 1, limited to the superficial epidermal and dermal layers. Includes redness that does not blanch to the touch and redness that requires intervention; 2, involving the epidermal and dermal layers and extending into the adipose tissue; 3, extending through superficial structures and adipose tissue down to and including muscle; 4, destruction of all soft tissue structures and communication with bone or joint structures.

### Neurological Recovery: Primary Outcome, ASIA Motor Score

Linear mixed models with ASIA motor score as the dependent variable demonstrated an association of PUs with less motor recovery. Differences in ASIA motor score between the unexposed and exposed groups were already present at discharge (median [IQR], 81 [56-117] days) and demarcate further at the primary end point 1 year (median [IQR], 13 [12-15] months) after SCI (Table 3). The estimated mean difference in ASIA motor score recovery between the exposed group and the unexposed reference group in the model adjusted for baseline only (model 1) was −6.3 points (95% CI, −8.6 to −3.9 points; *P* < .001) at discharge and −12.2 points (95% CI, −15.1 to −9.3 points; *P* < .001) at 1 year. Additional adjustment for sociodemographic variables and NLI (model 2) changed this association slightly, resulting in a −13.0-point difference (95% CI, −16.4 to −9.6 points; *P* < .001) between the groups at 1 year. After additional adjustment for the AIS that is a major predictor of outcome (model 3), the estimated difference was lower at −9.1 points (95% CI, −12.3 to −6.0 points; *P* < .001). After stratification of model 2 for AIS grade (models 4A, 4B, and 4C), the differences between the exposed and unexposed groups remained consistent across the strata but were smaller in the AIS A stratum compared with AIS B and C strata (Table 3). To account for a possible bias due to missing covariate data and/or attrition, recalculation of the model of main relevance (model 3) after multiple imputation of missing values in a sensitivity analysis (eTable 6 in Supplement 1) resulted in an estimated difference in ASIA motor score recovery between the exposed and unexposed groups of −6.7 points (95% CI, −9.9 to −3.4 points; *P* < .001).

**Table 3.** Association of Hospital-Acquired Pressure Ulcers (PUs) With American Spinal Injury Association Motor Score^a^

Model	Discharge (median [IQR] 81 [56-117] days after SCI)			1 y (median [IQR] 13 [12-15] months after SCI)			PU × Time	
	Patients, No.	Exposed vs unexposed, difference (95% CI)	*P* value	Patients, No.	Exposed vs unexposed, difference (95% CI)	*P* value	Change in difference between exposed and unexposed from discharge to 1 y (95% CI)	*P* value
1	1157	−6.3 (−8.6 to −3.9)	<.001	478	−12.2 (−15.1 to −9.3)	<.001	−5.9 (−8.3 to −3.5)	<.001
2	803	−7.2 (−10.0 to −4.5)	<.001	381	−13.0 (−16.4 to −9.6)	<.001	−5.8 (−8.6 to −3.0)	<.001
3	803	−4.1 (−6.6 to −1.6)	.001	381	−9.1 (−12.3 to −6.0)	<.001	−5.0 (−7.8 to −2.2)	.001
4A	451	−1.9 (−4.6 to 0.8)	.16	221	−5.9 (−9.3 to −2.5)	<.001	−4.0 (−6.9 to −1.0)	.01
4B	151	−9.5 (−16.7 to −2.4)	.01	70	−13.6 (−22.7 to −4.4)	.004	−4.0 (−11.4 to 3.6)	.32
4C	201	−5.3 (−11.4 to 0.8)	.09	90	−14.3 (−23.0 to −5.7)	.001	−9.0 (−17.1 to −0.8)	.04

Abbreviation: SCI, spinal cord injury.

^a^ Linear mixed regression models of American Spinal Injury Association (ASIA) motor score change from baseline. The table indicates the differences in ASIA motor score recovery between the exposed and unexposed groups at discharge from inpatient rehabilitation and 1 year follow-up expressed as difference of estimated marginal means with 95% CI and *P* value. In addition, the change of the estimated differences between the groups from discharge from inpatient rehabilitation to 1 year is reported (PU interaction with time). The models were adjusted for ASIA motor score at baseline (model 1), additionally adjusted for sociodemographic variables (age, sex, educational status, language status, and occupational status) and neurological level of injury (model 2), and subsequently further adjusted for AIS (model 3). In a sensitivity analysis, model 2 was stratified for ASIA impairment scale (AIS) (models 4A, 4B, and 4C). Center is considered a random effect for all models, except for model 4B due to convergence problems. No. describes number of patients with complete information on selected model variables at discharge or 1 year, respectively. Goodness of fit expressed as conditional *R*^2^ (*R*^2^c for fixed and random effects) and marginal *R*^2^ (*R*^2^m for fixed effects): model 1: *R*^2^c = 0.89, *R*^2^m = 0.54; model 2: *R*^2^c = 0.89, *R*^2^m = 0.56; model 3: *R*^2^c = 0.89, *R*^2^m = 0.66; model 4A: *R*^2^c = 0.84, *R*^2^m = 0,50; model 4B: *R*^2^c = 0.80, *R*^2^m = 0.30; model 4C: *R*^2^c = 0.72, *R*^2^m = 0.39.

### Secondary Neurological Outcomes: UEMS, LEMS, and AIS-Grade-Conversion

Associations of PUs with worse motor recovery were also evident for the UEMS and LEMS subscores of the ASIA motor score (eFigure 3, eTables 7 and 8 in Supplement 1). Changes toward a better in neurological injury class (AIS-conversion) were less frequent in the exposed group compared with the unexposed group at 1 year (eFigures 4 and 5 in Supplement 1).

### Functional Recovery: FIM Motor Score

The difference in FIM motor score recovery between the exposed and unexposed groups estimated by the linear mixed model adjusted for baseline FIM motor score (model 1) was −13.8 points (95% CI, −16.5 to −11.1 points; *P* < .001) at 1 year (Table 4). Further adjustment for sociodemographic variables and neurological level (model 2) barely changed this estimate. In the model additionally adjusted for the AIS (model 3), the estimated difference at 1 year was lower at −8.3 points (95% CI, −11.1 to −5.5 points; *P* < .001). In model 2, stratified for AIS (model 4A, 4B, 4C), the estimated differences remained consistent across the AIS-strata (Table 4). For explorative comparison of unadjusted FIM motor score recovery, see eFigure 6 in Supplement 1. The FIM subscore recovery was also different between the exposed and unexposed groups (eTable 9 in Supplement 1).

**Table 4.** Association of Hospital-Acquired Pressure Ulcers (PUs) With Functional Independence Measure Motor Scores^a^

Model	Discharge [81 (56-117) days after SCI]			1 y [13 (12-15) months after SCI]			PU × Time	
	Patients, No.	Exposed vs unexposed, difference (95% CI)	*P* value	Patients, No.	Exposed vs unexposed, difference (95% CI)	*P* value	Change in difference between exposed and unexposed from discharge to 1 y (95% CI)	*P* value
1	1157	−9.0 (−11.4 to −6.7)	<.001	674	−13.8 (−16.5 to −11.1)	<.001	−4.8 (−7.0 to −2.5)	<.001
2	819	−8.9 (−11.7 to −6.2)	<.001	579	−14.3 (−17.3 to −11.3)	<.001	−5.4 (−7.9 to −3.0)	<.001
3	819	−5.1 (−7.6 to −2.6)	<.001	579	−8.3 (−11.1 to −5.5)	<.001	−3.2 (−5.6 to −0.8)	.01
4A	453	−2.9 (−5.7 to −0.1)	.04	311	−6.8 (−10.0 to −3.7)	<.001	−3.9 (−6.8 to −1.1)	.01
4B	158	−7.0 (−13.3 to −0.7)	.03	116	−10.6 (−17.4 to −3.7)	.003	−3.6 (−8.9 to 1.7)	.20
4C	208	−8.2 (−14.4 to −1.9)	.01	152	−9.7 (−16.5 to −2.8)	.01	−1.5 (−7.4 to 4.3)	.62

Abbreviation: SCI, spinal cord injury.

^a^ Linear mixed regression models of the Functional Independence Measure (FIM) motor score change from baseline. The difference in FIM motor score recovery between the exposed and unexposed groups is expressed as difference of estimated marginal means with 95% CI and *P* value at discharge from inpatient rehabilitation and 1 year follow-up. In addition, the interaction effect of PU with time is reported. The models were adjusted for the FIM motor score at baseline (model 1), additionally adjusted for sociodemographic variables (age, sex, educational status, language status, and occupational status) and neurological level of injury (model 2), and subsequently further adjusted for American Spinal Injury Association impairment scale (AIS) (model 3). In a sensitivity analysis, the models were stratified for AIS (models 4A, 4B, and 4C). Center is considered a random effect for all models, except for model 4B due to convergence problems. No. describes number of patients with complete information on selected model variables at discharge or 1 year. Goodness of fit expressed as conditional *R*^2^ (*R*^2^c for fixed and random effects) and marginal *R*^2^ (*R*^2^m for fixed effects): model 1: *R*^2^c = 0.81, R^2^m = 0.37; model 2: *R*^2^c = 0.82, *R*^2^m = 0.39; model 3: *R*^2^c = 0.84, *R*^2^m = 0.52; model 4A: *R*^2^c = 0.78, *R*^2^m = 0.43; model 4B: *R*^2^c = 0.83, *R*^2^m = 0.41; model 4C: *R*^2^c = 0.78, *R*^2^m = 0.40.

### Post Hoc Analyses: Associations With Period of First Occurrence of PUs and Combined Exposure PUs and Infections

The period of medical care at the first occurrence of PUs was not associated with the neurological or functional outcome at 1 year. PUs acquired in both acute care and inpatient rehabilitation were associated with poorer recovery compared with the unexposed group, and their outcome was not different (eFigure 7 in Supplement 1).

The examination of potential additive associations of PUs and hospital acquired infections revealed that patients who selectively acquired either a PU or an infection experienced lower gains in ASIA motor score, UEMS, LEMS, and FIM motor score at 1 year compared with patients without PUs or infections. Patients acquiring both PUs and infections had the worst neurological and functional outcomes (eFigure 8 in Supplement 1).

### PU-Associated Long-Term Mortality

Survival analysis up to the 10-year follow-up demonstrated a higher cumulative mortality in the exposed group compared with the unexposed group. Similar to the unadjusted Kaplan-Meier curves (eFigure 9 in Supplement 1), Cox regression adjusted for neurological level and sociodemographic variables demonstrated that PUs were associated with a higher mortality risk (hazard ratio [HR], 1.80; 95% CI, 1.41-2.31; *P* < .001). This was confirmed after adjustment for the AIS (HR, 1.41; 95% CI, 1.09-1.82; *P* = .01). When stratified for the individual AIS grades, the positive association of PUs with mortality was visible in all strata, although less clearly in the smaller AIS B and C strata (Table 5).

**Table 5.** Cox Regression Model of Mortality Risk up to 10 Years After Spinal Cord Injury Associated With Pressure Ulcer^a^

Model	Patients, No.	HR (95% CI)	*P* value
1	1241	1.62 (1.29 to 2.05)	<.001
2	1094	1.80 (1.41 to 2.31)	<.001
3	1094	1.41 (1.09 to 1.82)	.01
4A	597	1.36 (0.99 to 1.87)	.06
4B	220	1.50 (0.79 to 2.85)	.21
4C	277	1.35 (0.73 to 2.48)	.34

Abbreviation: HR, Hazard Ratio.

^a^ Cox proportional hazards models of mortality up to 10 years with random intercept for center: unadjusted (model 1), adjusted for sociodemographic variables (age, sex, occupational status) and for neurological level of injury (model 2). Subsequently the model was further adjusted for American Spinal Injury Association impairment scale (AIS) (model 3). The model 2 was also calculated stratified for AIS (models 4A, 4B, and 4C).

## Discussion

We report the first multicenter cohort study we know of to reveal and quantify the association of acquired PUs with (1) neurological recovery and (2) functional independence at 1 year and (3) survival up to 10 years after SCI. Adjusted linear mixed models revealed a robust association of PUs acquired during initial hospitalization after cervical SCI with poorer neurological long-term recovery. Confounder adjusted estimated differences (model 3) between the unexposed and exposed group of −9.1 ASIA motor score points after 1 year in the available cases and −6.7 points after multiple imputation of missing values are clinically relevant^29^ and within the 5 to 10 ASIA motor score point recovery range (from baseline) considered to be the efficacy end point in interventional trials.^23^ Secondary outcome analyses assessing upward AIS-conversions as a measure of mixed sensory-motor function confirmed fewer upward AIS-conversions in patients with acquired PUs. The functional relevance of these findings was corroborated by adjusted analysis revealing a negative association of PUs with the recovery of functional independence (FIM motor score) at 1 year and with survival up to 10 years after SCI.

A long-term association of PU exposure with neurological and functional recovery is further supported by the fact that group differences evident at discharge from inpatient rehabilitation were less pronounced than differences after 1 year. In addition, a group difference in PU-associated mortality was also less evident initially, but increased over time in the Kaplan-Meier analysis. The PU association with outcome appeared to be dependent on at least some preserved recovery potential. The intrinsic recovery potential is larger in incomplete patients with SCI graded AIS B and C compared with complete AIS A patients.^23^ Correspondingly, in patients graded AIS B and C, the observed associations of PUs with motor and functional recovery were larger compared with AIS A. In absence of a greater recovery potential, the smaller difference in the AIS A group can be considered floorlike. Together, this supports that variable responses to rehabilitation, despite similar initial injury severity, could be attributed to PU-induced neuropathology, which may undermine the endogenous recovery potential. PUs modify the inflammatory tone systemically and thereby signal far beyond its localized appearance.^7,8,9,10^ Molecular mechanisms by which supernumeric inflammatory foci can propagate damage and blunt repair-programs (disrepair) have been delineated only recently.^12,19^

To gauge the PU association and investigate for other, nonredundant, possibly aggravating factors, we extended the analysis post hoc comparing the PU exposure with the event of acquired infections.^30,31,32^ Compared with acquired PUs alone, the development of an additional infection is associated with further worsened neurological and functional outcome in line with nonredundant, additive PU effects. These data provide evidence consistent with PUs as an independent factor associated with risk for poor neurological recovery (recovery confounder).

### Limitations

This study has limitations. A potential unmeasured confounder in this study is the individual patient’s health care utilization. However, the estimated recovery differences relatable to PUs barely changed after adjusting for sociodemographic baseline variables that may influence health care utilization. A clear reduction in differences associated with PUs was observed after adjusting for SCI severity (AIS), which is a strong predictor of recovery after SCI.^23^ In the models stratified for AIS, the associations of PUs were consistent across the strata. This emphasizes the robustness and plausibility of the results. The negative association of ASIA motor score recovery with PUs was confirmed using a multiple imputation technique as a sensitivity analysis. The estimate changed to some extent in only 1 subgroup (AIS B) in the stratified model at 1 year. This may reflect the higher uncertainty in this small subgroup rather than a relevant bias due to missing data.

## Conclusions

This cohort study suggests PUs qualify as modifiable disease-modifying factors after SCI. Considering patients with SCI to be at higher risk to develop PUs in nonspecialized SCI centers, these results emphasize the need to rethink referral patterns for patients with SCI. Focused referral directed to centers with established SCI care algorithms and knowledge dissemination^33^ can prevent PUs development and protect endogenous recovery potential.^34,35^ Despite increased translational efforts^36^ the outcome of SCI has not improved in recent decades.^37,38^ While agonistic experimental strategies to facilitate neuroaxonal repair remain crucial for developing novel treatment modalities in the future, clinical phenotypes of impaired recovery associated with PUs offer clinical opportunities to rectify patient stratification for interventional trials, preserve the endogenous recovery potential, and improve neurological and functional recovery.

## Notes

Supplement 1.
eMethods
**eFigure 1. **Dataset selection and analysis chart **eFigure 2.** Directed Acyclic Graph (DAG) considering potential relationships between variables **eFigure 3. **ASIA motor score changes during the first year after SCI **eFigure 4.** ASIA impairment scale (AIS)-conversion over the first year after SCI **eFigure 5.** Stepwise ASIA impairment scale (AIS)-conversion over the first year after SCI **eFigure 6. **FIM motor score changes from baseline over the first year after SCI **eFigure 7.** Association of PU onset with ASIA motor score and FIM motor score recovery after SCI **eFigure 8.** Individual versus combined associations of acquired infections and pressure ulcers with ASIA motor score and FIM motor score recovery after SCI **eFigure 9. **Effect of hospital-acquired pressure ulcers on survival up to 10 years **eTable 1.** Variable definitions for neurological classification of spinal cord injury according to the SCIMS data dictionary 2000 to 2005 **eTable 2.** Grades of pressure ulcer as defined in the SCIMS data-dictionary 2000 to 2005 **eTable 3.** Functional Independence Measure (FIM) score and subscores as defined in the SCIMS data-dictionary 2000 to 2005 **eTable 4.** Comparison of sociodemographic baseline characteristics of screened, included, and excluded patients **eTable 5. **Comparison of sociodemographic and baseline characteristics of complete cases with patients lost to follow-up **eTable 6. **Association of hospital-acquired pressure ulcers with ASIA motor recovery after Multiple Imputation **eTable 7. **Association of hospital-acquired pressure ulcers with UEMS recovery **eTable 8. **Association of hospital-acquired pressure ulcers with LEMS recovery **eTable 9.** Improvement in FIM subscores and total FIM motor score from baseline to 1 year follow-up
eReferences

Supplement 2.
Data Sharing Statement
