# Supplementary material for: Hospital-Acquired Pressure Ulcers and Long-Term Motor Score Recovery in Patients With Acute Cervical Spinal Cord Injury
Source: JAMA Netw Open. 2024 Dec 6;7(12):e2444983. doi: 10.1001/jamanetworkopen.2024.44983 (PMC11624580; doi:10.1001/jamanetworkopen.2024.44983)
Supplement: Supplement 1. — eMethods eFigure 1. Dataset selection and analysis chart eFigure 2. Directed Acyclic Graph (DAG) considering potential relationships between variables eFigure 3. ASIA motor score changes during the first year after SCI eFigure 4. ASIA impairment scale (AIS)-conversion over the first year after SCI eFigure 5. Stepwise ASIA impairment scale (AIS)-conversion over the first year after SCI eFigure 6. FIM motor score changes from baseline over the first year after SCI eFigure 7. Association of PU onset with ASIA motor score and FIM motor score recovery after SCI eFigure 8. Individual versus combined associations of acquired infections and pressure ulcers with ASIA motor score and FIM motor score recovery after SCI eFigure 9. Effect of hospital-acquired pressure ulcers on survival up to 10 years eTable 1. Variable definitions for neurological classification of spinal cord injury according to the SCIMS data dictionary 2000 to 2005 eTable 2. Grades of pressure ulcer as defined in the SCIMS data-dictionary 2000 to 2005 eTable 3. Functional Independence Measure (FIM) score and subscores as defined in the SCIMS data-dictionary 2000 to 2005 eTable 4. Comparison of sociodemographic baseline characteristics of screened, included, and excluded patients eTable 5. Comparison of sociodemographic and baseline characteristics of complete cases with patients lost to follow-up eTable 6. Association of hospital-acquired pressure ulcers with ASIA motor recovery after Multiple Imputation eTable 7. Association of hospital-acquired pressure ulcers with UEMS recovery eTable 8. Association of hospital-acquired pressure ulcers with LEMS recovery eTable 9. Improvement in FIM subscores and total FIM motor score from baseline to 1 year follow-up eReferences [file jamanetwopen-e2444983-s001.pdf]

Supplemental Online Content

Kopp MA, Finkenstaedt FW, Schweizerhof O, et al. Hospital-acquired pressure ulcers and long-term motor score recovery in patients with acute cervical spinal cord injury. *JAMA Netw Open*. 2024;7(11):e2444983. doi:10.1001/jamanetworkopen.2024.44983

eMethods
eFigure 1. Dataset selection and analysis chart
eFigure 2. Directed Acyclic Graph (DAG) considering potential relationships between variables
eFigure 3. ASIA motor score changes during the first year after SCI
eFigure 4. ASIA impairment scale (AIS)-conversion over the first year after SCI
eFigure 5. Stepwise ASIA impairmet scale (AIS)-conversion over the first year after SCI
eFigure 6. FIM motor score changes from baseline over the first year after SCI
eFigure 7. Association of PU onset with ASIA motor score and FIM motor score recovery after SCI
eFigure 8. Individual versus combined associations of acquired infections and pressure ulcers with ASIA motor score and FIM motor score recovery after SCI
eFigure 9. Effect of hospital-acquired pressure ulcers on survival up to 10 years
eTable 1. Variable definitions for neurological classification of spinal cord injury according to the SCIMS data dictionary 2000 to 2005
eTable 2. Grades of pressure ulcer as defined in the SCIMS data-dictionary 2000 to 2005
eTable 3. Functional Independence Measure (FIM) motor score and subscores as defined in the SCIMS data-dictionary 2000 to 2005
eTable 4. Comparison of sociodemographic baseline characteristics of screened, included, and excluded patients
eTable 5. Comparison of sociodemographic and baseline characteristics of complete cases with patients lost to follow-up
eTable 6. Association of hospital-acquired pressure ulcers with ASIA motor score recovery after Multiple Imputation
eTable 7. Association of hospital-acquired pressure ulcers with UEMS recovery
eTable 8. Association of hospital-acquired pressure ulcers with LEMS recovery
eTable 9. Improvement in FIM subscores and total FIM motor score from baseline to 1 year follow-up
eReferences

This supplemental material has been provided by the authors to give readers additional information about their work.

© 2024 Kopp MA et al. *JAMA Network Open.*

eMethods

Variable definitions

The severity of pressure ulcers (PUs) (eTable 2) and their locations and number were documented during acute surgical care and inpatient rehabilitation after acute traumatic spinal cord injury (SCI) in a standardized manner throughout the observation period. The onset of a PU or a worsening of an existing PU must have been occurred during these intervals. PUs of any severity (ie, grade 1 to 4) documented at least once during acute surgical care and/or inpatient rehabilitation were combined as a dichotomized exposure variable. In order to explore outcome differences in relation to the period of first occurrence of the PU, we further categorized the PU variable as 'no PU', 'first PU during acute care', and 'first PU during inpatient rehabilitation'. For the exploration of individual and combined relationships of the factors 'PU' and 'infection'[1,2] the Spinal Cord Injury Model Systems variables 'pneumonia' and 'postoperative wound infection' were considered as defined earlier[2] and categorized as 'no infection or PU', 'PU only', 'infection only', and 'infection and PU'.

The International Standards for Neurological Classification of Spinal Cord Injury (ISNCSCI) were applied for neurological assessments utilizing the items: American Spinal Injury Association (ASIA) impairment scale (AIS), the ASIA motor score and its Upper Extremity Motor Score (UEMS) and Lower Extremity Motor Score (LEMS) subscales. The ASIA motor score, UEMS, and LEMS were calculated from the grade of strength of the respective key muscles (eTable1). The Functional Independence Measure (FIM) motor items that assess the physical independence in key actions of daily life were collected directly by study personnel or structured interviews. The FIM motor score and four FIM subscores were computed from the 13 single FIM motor items (eTable 3).

Neurological and functional outcome data were collected at admission to acute surgical care and/or inpatient rehabilitation (baseline), at discharge from inpatient rehabilitation (discharge), and at the first annual examination after SCI (1 year). In case that the ISNCSCI examination data was incomplete at admission to surgical care, missing data were replaced with the INSCSCI data collected at admission to inpatient rehabilitation. The FIM baseline data was collected at admission to rehabilitation only. Differences between ASIA motor score, UEMS, LEMS, or FIM motor score values at baseline to the respective values at follow-up were calculated for statistical analysis. Change in the AIS (AIS-conversion) from baseline to 1 year was analyzed broken down in two categories (improved vs. stable or worsened) as well as in the individual grades of AIS-change.

The survival status of the study participants after discharge from inpatient rehabilitation was assessed using information collected through various mechanisms such as clinical records, obituary, returned mail, and regularly performed social security death index queries. Survival

© 2024 Kopp MA et al. *JAMA Network Open.*

time was calculated as the interval between the date of injury to the date of death, censoring at 10 years after SCI at latest.

Multiple Imputation

For imputation, we used multivariate imputation by chained equation (mice package in R)[3] under the missing at random assumptions with m=30 datasets. Depending on the scale of variable that contained missings we used different imputation methods (`pmm` for continuous and `logreg` for binary variables). To account for repeated measurements, imputation was done in wide format. To consider interactions of variables with the PU variable, data for patients with PUs and without PUs were imputed separately. We included motor scores measured at different time points after SCI (UEMS and LEMS at admission to acute care, admission to inpatient rehabilitation, discharge from inpatient rehabilitation, 1 year; FIM motor score at admission to inpatient rehabilitation, discharge from inpatient rehabilitation, 1 year, and 5 years). The total ASIA motor score was derived passively by calculation after imputation (ASIA motor score = UEMS + LEMS). Further, we incorporated information on neurological level of injury (C1-C4 vs. C5-C8), AIS (A, B, C), penetrating injury (yes vs. no), age, sex, demographics, work status (unemployed/retired vs. working/studying), all obtained from the acute care period. Information on acquired pneumonia (yes vs. no) and wound infection (yes vs. no) were considered during both acute care and inpatient rehabilitation periods. To include information on death, we approximated the cumulative risk within ten years using the Nelson-Aalen estimator and used this information in addition to the event indicator variable in the imputation procedure (QU).[4] All results shown with multiple imputed datasets were pooled according to Rubin`s Rules and the marginal R^2 and conditional R^2 represent the mean of the imputed datasets.

© 2024 Kopp MA et al. *JAMA Network Open.*

eFigure 1. Dataset selection and analysis chart.

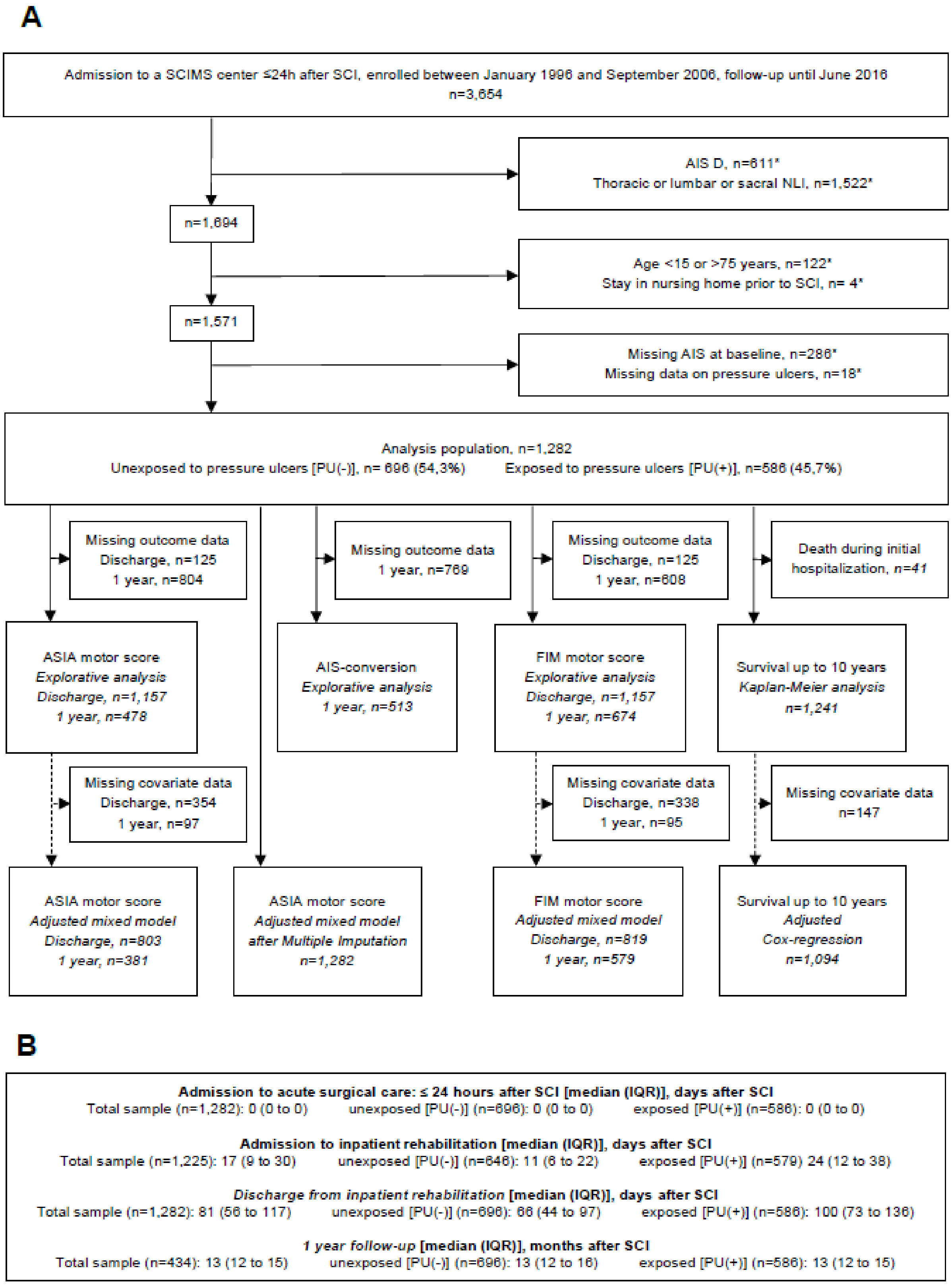

eFigure 1: (A) Patients admitted to a SCIMS center within 24 hours after SCI were assigned to long-term follow-up. Datasets collected between 1996 and 2006 comprise data on PUs, ASIA motor score, and FIM motor score outcome. The data selection for statistical analysis considered possible confounding factors, i.e. ceiling effects in mild SCI (AIS D) or age extremes as well as the availability of

© 2024 Kopp MA et al. *JAMA Network Open.*

key variables, that is PU and neurological baseline data. *Total numbers of excluded cases may differ from subgroup numbers because some items applied multiple times. **(B)** Assessment times following SCI are indicated as medians and interquartile range. Abbreviations: AIS = ASIA impairment scale, ASIA = American Spinal Injury Association, FIM = Functional Independence Measure, PU = pressure ulcer, NLI = Neurological level of injury, SCI = spinal cord injury, SCIMS = SCI Model Systems.

© 2024 Kopp MA et al. *JAMA Network Open.*

eFigure 2. Directed Acyclic Graph (DAG) considering potential relationships between variables

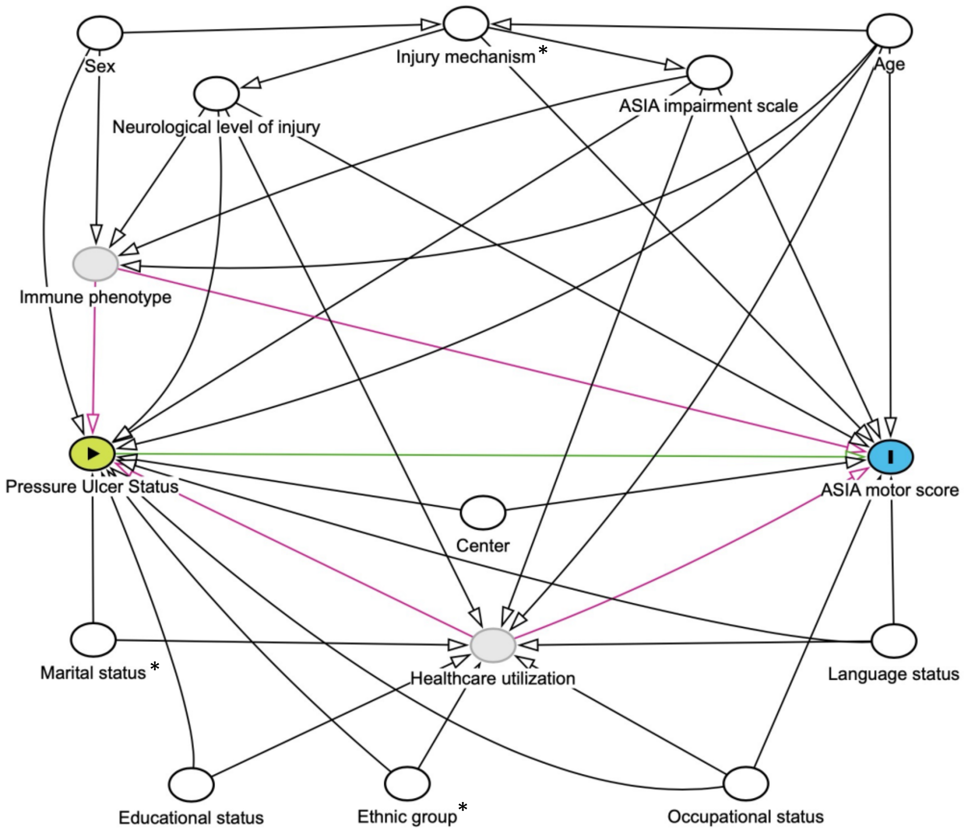

eFigure 2: Directed Acyclic Graph (DAG) to visualize relationships (arrows) between variables taken into consideration for the linear mixed model calculated to reveal associations of the exposure pressure ulcer (PU) (green node) on the primary outcome ASIA motor score (blue node), as well as to depict 'hypothetical causal pathways' (green arrow) and 'potential biasing pathways' (red arrows) due to unmeasured confunders. Potential unmeasured confounders are the immune phenotype and the individual patient's healthcare utilization. The immune phenotype may affect both PU status[5] and neurological outcome.[6] However, the model was adjusted for the SCI severity (AIS), the neurological level of injury, and key demographic variables (age and sex), all of which can have colliding effects on the immune phenotype or on neurological outcome.[7-9] The utilization of inpatient and follow-up healthcare as an additional counfounder could not be addressed directly, but some sociodemographic factors (i.e., occupational status, language status, or educational status) that may influence the degree of healthcare utilisation were included in the models. The decision for inclusion of the variables was based on the Akaike information criterion (AIC). Other available variables considered during model development (indicated by asterisks) were injury mechanism, ethnic group, and marital status. Because these variables did not contribute to the predictive value of the model, they were excluded from the final model as it can be assumed that these variables have no relevant biasing effect. The graph was drawn using the online application of the DAGitty Software[10] Version 3.1 (URL: http://www.dagitty.net/dags.html). Abbreviations: AIC = Akaike Information Criterion, AIS = ASIA impairment scale, ASIA = American Spinal Injury Association, PU = pressure ulcer.

© 2024 Kopp MA et al. *JAMA Network Open.*

eFigure 3. ASIA motor score changes during the first year after SCI

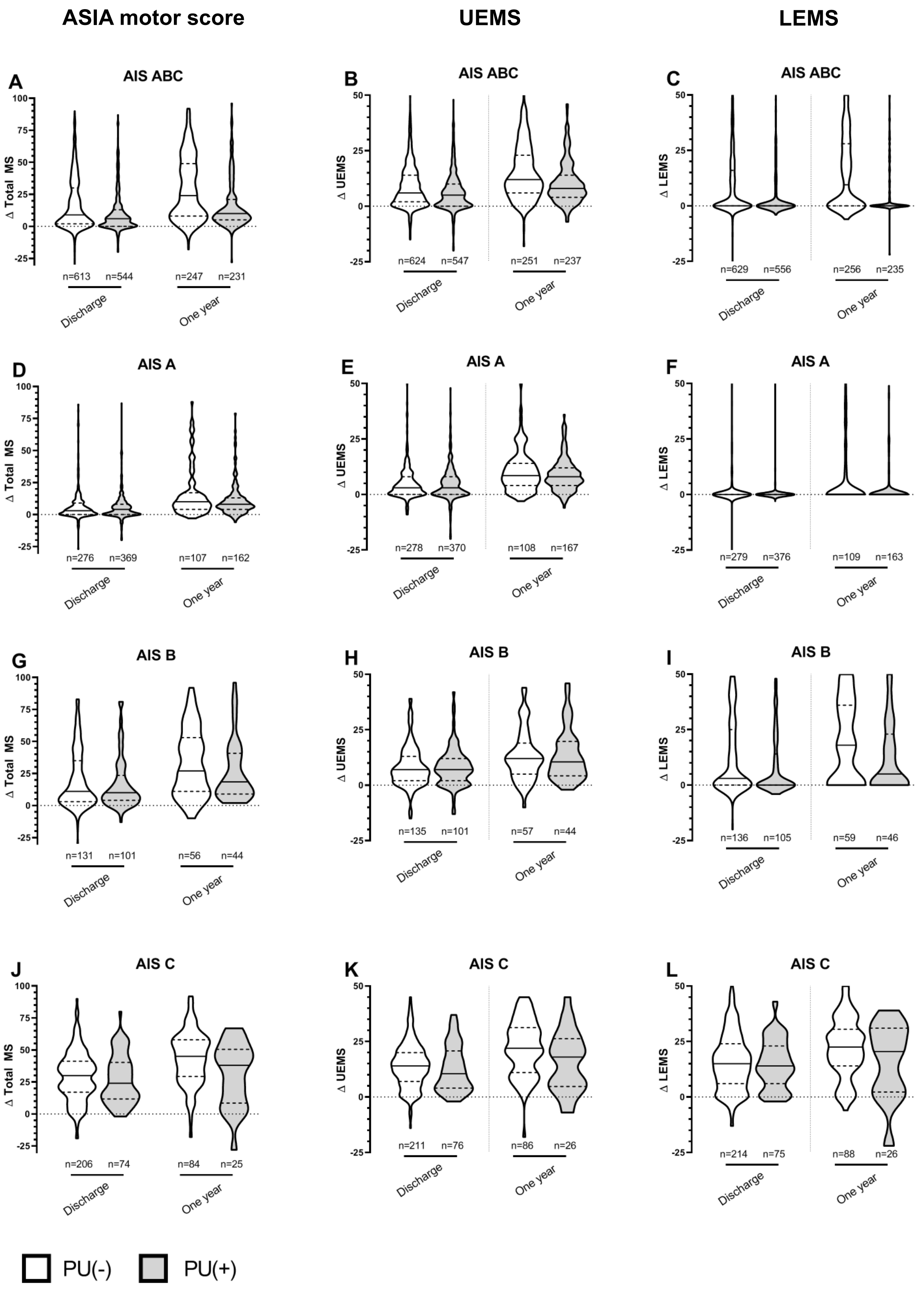

eFigure 3: ASIA motor score changes over time in **(A-C)** the total sample (AIS A, B, C) and **(G-L)** the respective individual AIS strata (AIS A vs. B vs. C) comparing patients unexposed to pressure ulcers [PU(-)] with patients exposed to pressure ulcers [PU(+)] at discharge [81 (56 to 117) days after SCI,

© 2024 Kopp MA et al. *JAMA Network Open.*

median, (interquartile range)] and one year follow-up [13 (12 to 15) months after SCI, median (interquartile range)]. The ASIA motor score and its subscores are part of the International Standards for Neurological Classification of Spinal Cord Injury (ISNCSCI)[11] and represent the ISNCSCI component, which is most sensitive for changes.[12] UEMS and LEMS have improved correlation with functional outcomes.[13] **(D-F)** In the most severely injured SCI patients (AIS A) the differences to baseline in the PU(-) group scattered more upward than in the PU(+) group, especially for the LEMS. Here, the values are mostly at zero or close to zero. In the AIS A stratum, characterized by minor endogenous recovery potential,[8] also smaller differences between PU(+) and PU(-) in the recovery of ASIA motor score or UEMS were observed compared with **(G-I)** incomplete SCI AIS B and **(J-L)** AIS C, for which a larger recovery potential has been reported. **(F)** Group differences in improvement in LEMS are based mainly on differences in spread in the AIS A stratum as median and quartiles were zero**.** Groups with and without PUs were compared using the Mann-Whitney test adjusted for repeated measures over time according to Bonferroni. Total sample AIS A, B, C: **(A)** ASIA motor score, discharge $p < 0.001$, one year $p < 0.001$; **(B)** UEMS, discharge $p < 0.001$, one year p= $p < 0.001$; **(C)** LEMS, discharge $p < 0.001$, one year $p < 0.001$. Stratum AIS A: **(D)** ASIA motor score, discharge $p = 1.0$, one year $p = 0.13$; **(E)** UEMS, discharge $p = 1.0$, one year $p = 0.56$; **(F)** LEMS, discharge $p = 0.029$, one year $p = 0.009$. Stratum AIS B: **(G)** ASIA motor score, discharge $p = 0.75$, one year $p = 0.40$; **(H)** UEMS, discharge $p = 1.0$, one year $p=1.0$; **(I)** LEMS, discharge $p = 0.053$, one year $p = 0.12$. Stratum AIS C: **(J)** ASIA motor score, discharge $p = 0.50$, one year $p = 0.054$; **(K)** UEMS, discharge $p = 0.29$, one year $p=0.048$; **(L)** LEMS, discharge $p=1.0$, one year $p=0.36$. Median/quartiles are indicated as solid/broken lines, respectively. Abbreviations: AIS = ASIA impairment scale, ASIA = American Spinal Injury Association, FIM = Functional Independence Measure, LEMS = Lower Extremity Motor Score, MS = Motor Score, PU = pressure ulcer, UEMS = Upper Extremity Motor Score.

© 2024 Kopp MA et al. *JAMA Network Open.*

eFigure 4. ASIA impairment scale (AIS)-conversion over the first year after SCI

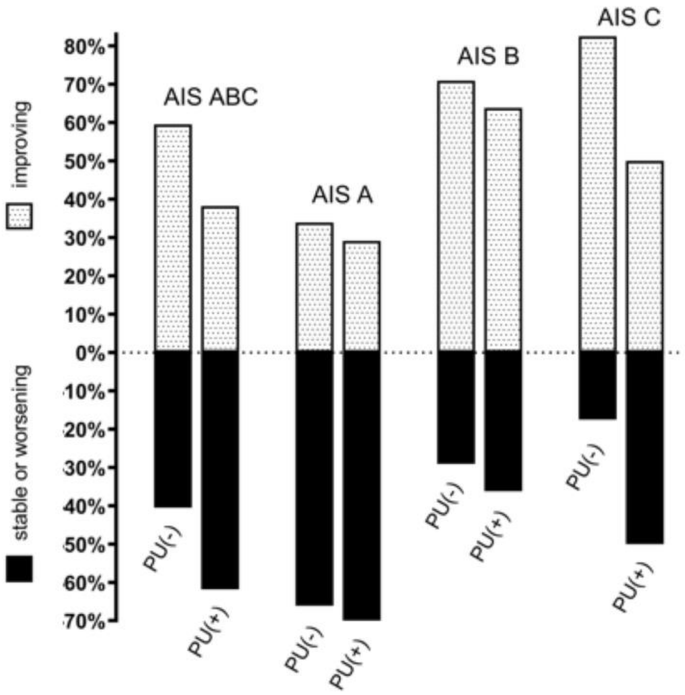

eFigure 4: AIS-conversion during the first year [13 (12 to 15) months after SCI, median (interquartile range)] in the total sample (AIS A, B, C) and respective individual AIS strata/severities (A vs. B vs. C) comparing patients unexposed to pressure ulcers [PU(-)] with patients exposed to pressure ulcers [PU(+)]. The AIS is a classification of injury completeness taking motor and sensory function into account.[11] The total sample (AIS A, B, C) revealed less frequent AIS-conversions (n = 95 of 249; 38.2%) in the PU(+) compared with the PU(-) group (n = 157 of 264; 59,5%). When stratified by AIS at baseline, the conversion rate is [PU(+) vs. PG(-); n = 50 of 172; 29.1% vs. n = 38 of 112; 33.9%] in the AIS A; n = 30 of 47; 63.8%, vs. n = 39 of 55; 70.9%) in the AIS B, and n = 15 of 30; 50.0%, vs. n = 80 of 97; 82.5% in the AIS C stratum. Groups with and without PUs were compared using the Chi-square test: AIS A, B, C p < 0.001; AIS A p = 0.43; AIS B p = 0.53; AIS C p = 0.001. Abbreviations: AIS = ASIA impairment scale, ASIA = American Spinal Injury Association, PU = pressure ulcer.

© 2024 Kopp MA et al. *JAMA Network Open.*

eFigure 5. Stepwise ASIA impairmet scale (AIS)-conversion over the first year after SCI

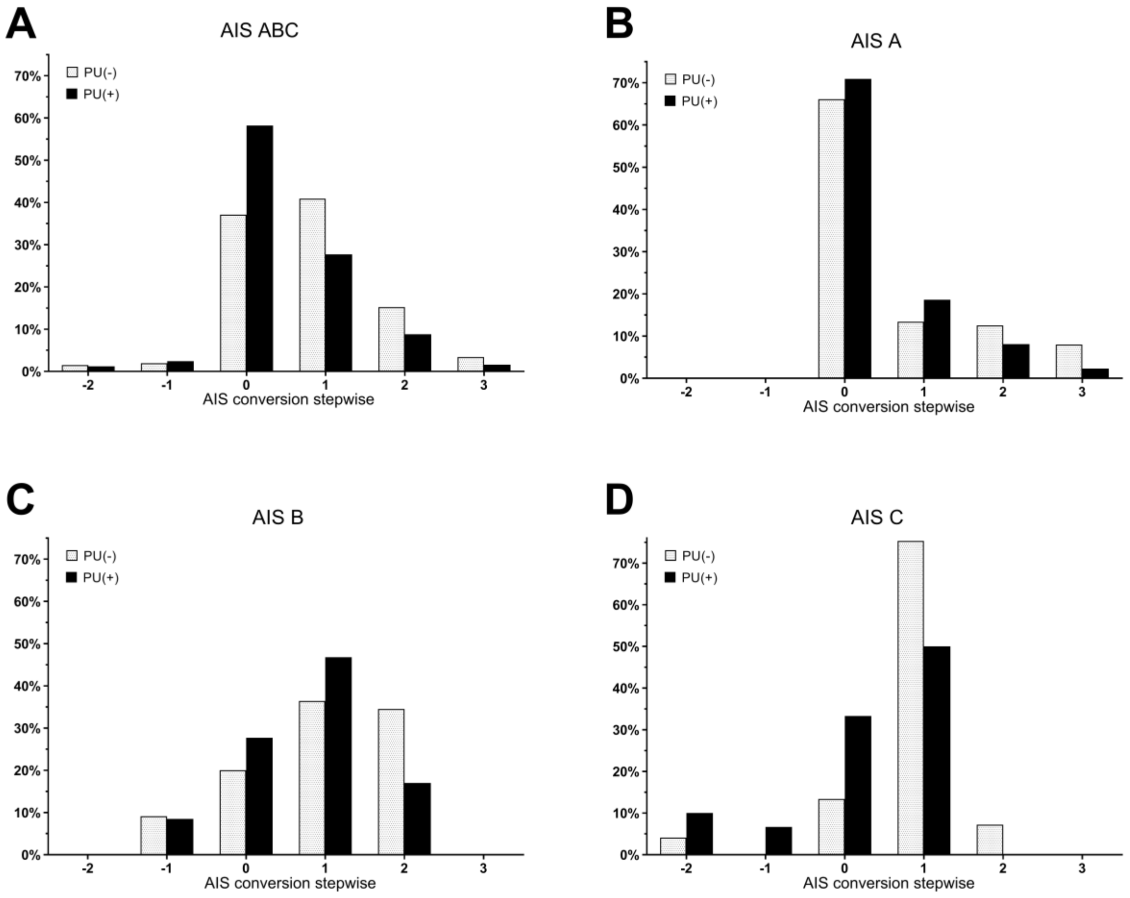

eFigure 5: Stepwise AIS-conversion in the total sample (AIS A, B, C) and the respective individual baseline AIS strata/severities (AIS A vs. B vs. C) comparing patients unexposed to pressure ulcers [PU(-)] with patients exposed to pressure ulcers [PU(+)] at 1 year follow-up [13 (12 to 15) months after SCI, median (interquartile range)]. The analysis of the AIS-conversions during the first year after SCI broken down by quantity of change reveals a differential pattern allowing to indentify subgroups displaying "marked recovery" (improvement of at least two AIS-grades).[14,15] **(A)** In the total sample, the initial lesion severity (AIS) more frequently remained unchanged and conversion rates to better AIS classifications were lower in patients exposed to PUs. In reverse, SCI patients without PUs improved proportionally more often in the AIS by one or more grades demonstrating higher conversion rates also with regards to "marked recovery". **(B–D)** In the strata AIS A and AIS B, patients with PUs revelad less frequently a "marked recovery" by to or three grades compared to patients without PUs. In the stratum AIS C, patients without PUs were more frequently improving by one or two AIS grades compared to patients with PUs, who demonstrated more often a worsening in the AIS by one or two grades. Sample size: AIS A, B, C = 513, AIS A = 284, AIS B = 102, AIS C = 127. Abbreviations: AIS = ASIA impairment scale, ASIA = American Spinal Injury Association, PU = pressure ulcer.

© 2024 Kopp MA et al. *JAMA Network Open.*

eFigure 6. FIM motor score changes from baseline over the first year after SCI

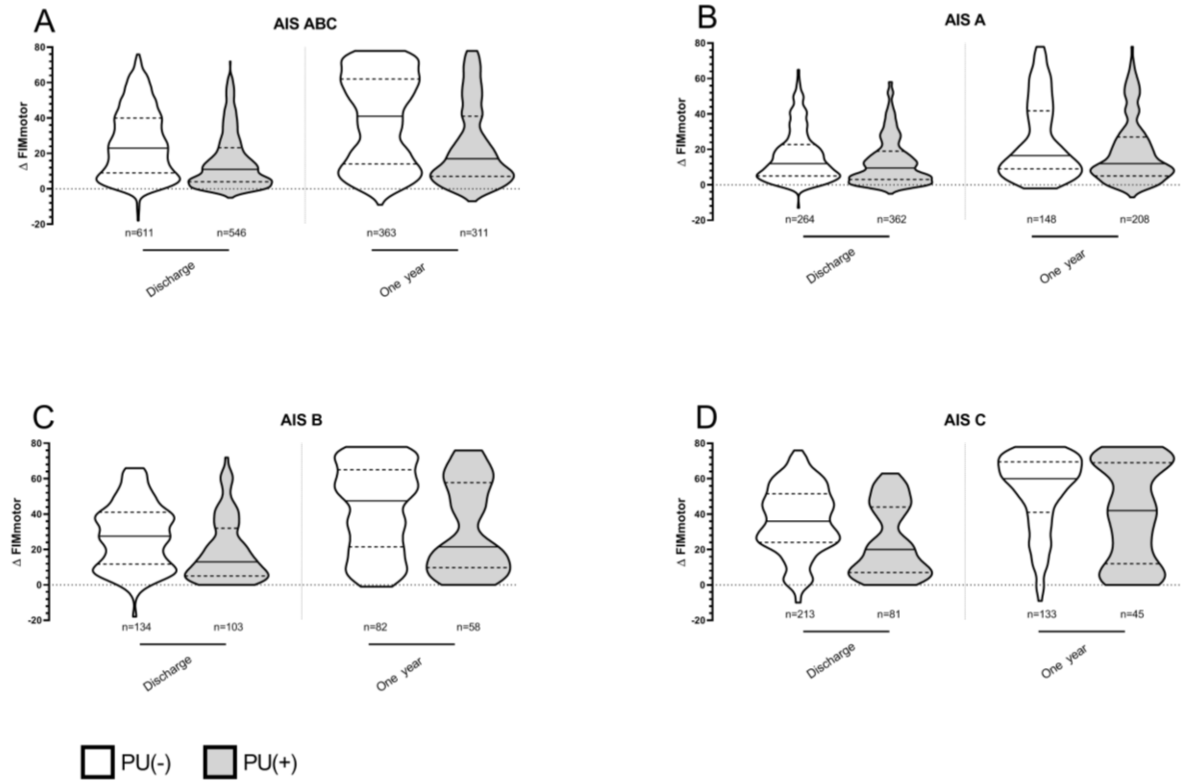

eFigure 6: FIM motor score changes from baseline over time in the total sample (AIS A, B, C) and the respective individual AIS strata (A vs. B vs. C) comparing patients unexposed to pressure ulcers [PU(-)] with patients exposed to pressure ulcers [PU(+)] at discharge [81 (56 to 117) days after SCI, median, (interquartile range)] and one year follow-up [13 (12 to 15) months after SCI, median (interquartile range)]. FIM motor score indicates functional recovery and the ability to participate in activities of daily living and correlates well with the Spinal Cord Independence Measure (SCIM).[16] The violin plots are displaying the FIM motor score changes as a difference from the baseline score collected at admission to inpatient rehabilitation to i) discharge and ii) 1 year after SCI in **(A)** the total sample (AIS A, B, C) and **(B-D)** the AIS strata. Groups with and without PUs were compared using the Mann-Withney test adjusted for repeated measures over time according to Bonferroni: **(A)** AIS A, B, C discharge $p < 0.001$, one year $p < 0.001$; **(B)** AIS A discharge $p = 0.002$, one year $p = 0.029$**; (C)** AIS B discharge $p < 0.001$, one year $p = 0.027$; **(D)** AIS C discharge $p < 0.001$; one year $p = 0.033$. Median/quartiles are indicated as solid/broken lines. Abbreviations: AIS = ASIA impairment scale, ASIA = American Spinal Injury Association, FIM = Functional Independence Measure, PU = pressure ulcer.

© 2024 Kopp MA et al. *JAMA Network Open.*

eFigure 7. Association of PU onset with ASIA motor score and FIM motor score recovery after SCI

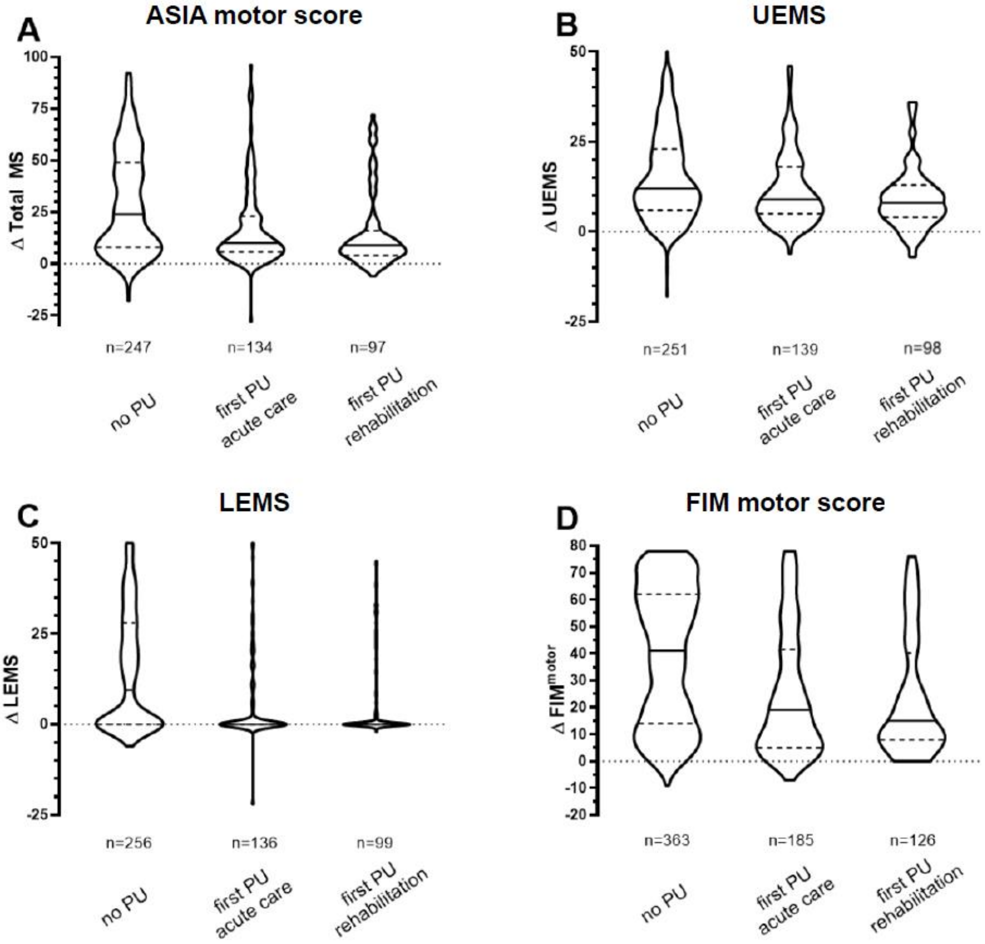

eFigure 7: Association of the period of first occurence of PUs with neurological and functional outcome. Both groups exposed to PUs (first PU during acute care and first PU during inpatient rehabilitation) revealed lower gains in motor and functional outcome scores compared with the unexposed group without PUs, but were not distinct from each other. The violin plots indicate **(A)** ASIA motor score, **(B)** UEMS, **(C)** LEMS**,** and **(D)** FIM motor score differences from baseline to 1 year [13 (12 to 15) months after SCI, median (interquartile range)] in the total sample (AIS A, B, C). Groups were compared using the Kruskal-Wallis test with Bonferroni adjusted post-tests. **(A)** ASIA motor score p < 0.001; post-tests: ‘no PU’ vs. first PU acute care’ p < 0.001, ‘no PU’ vs. ‘first PU rehabilitation’ p < 0.001, ‘first PU acute care’ vs. ‘first PU rehabilitation’ p = 0.74. **(B)** UEMS p < 0.001; post-tests: ‘no PU’ vs. first PU acute care’ p = 0.01, ‘no PU’ vs. ‘first PU rehabilitation’ p < 0.001, ‘first PU acute care’ vs. ‘first PU rehabilitation’ p = 0.26. **(C)** LEMS p < 0.001; post-tests: ‘no PU’ vs. first PU acute care’ p < 0.001, ‘no PU’ vs. ‘first PU rehabilitation’ p < 0.001, ‘first PU acute care’ vs. ‘first PU rehabilitation’ p = 1.0. **(D)** FIM motor score p < 0.001; post-tests: ‘no PU’ vs. first PU acute care’ p < 0.001, ‘no PU’ vs. ‘first PU rehabilitation’ p < 0.001, ‘first PU acute care’ vs. ‘first PU rehabilitation’ p = 1.0. Median/quartiles are indicated as solid/broken lines. Abbreviations: AIS = ASIA impairment scale, ASIA = American Spinal Injury Association, FIM = Functional Independence Measure, LEMS = Lower Extremity Motor Score, MS = Motor Score, PU = pressure ulcer, UEMS = Upper Extremity Motor Score.

© 2024 Kopp MA et al. *JAMA Network Open.*

eFigure 8. Individual versus combined associations of acquired infections and pressure ulcers with ASIA motor score and FIM motor score recovery after SCI

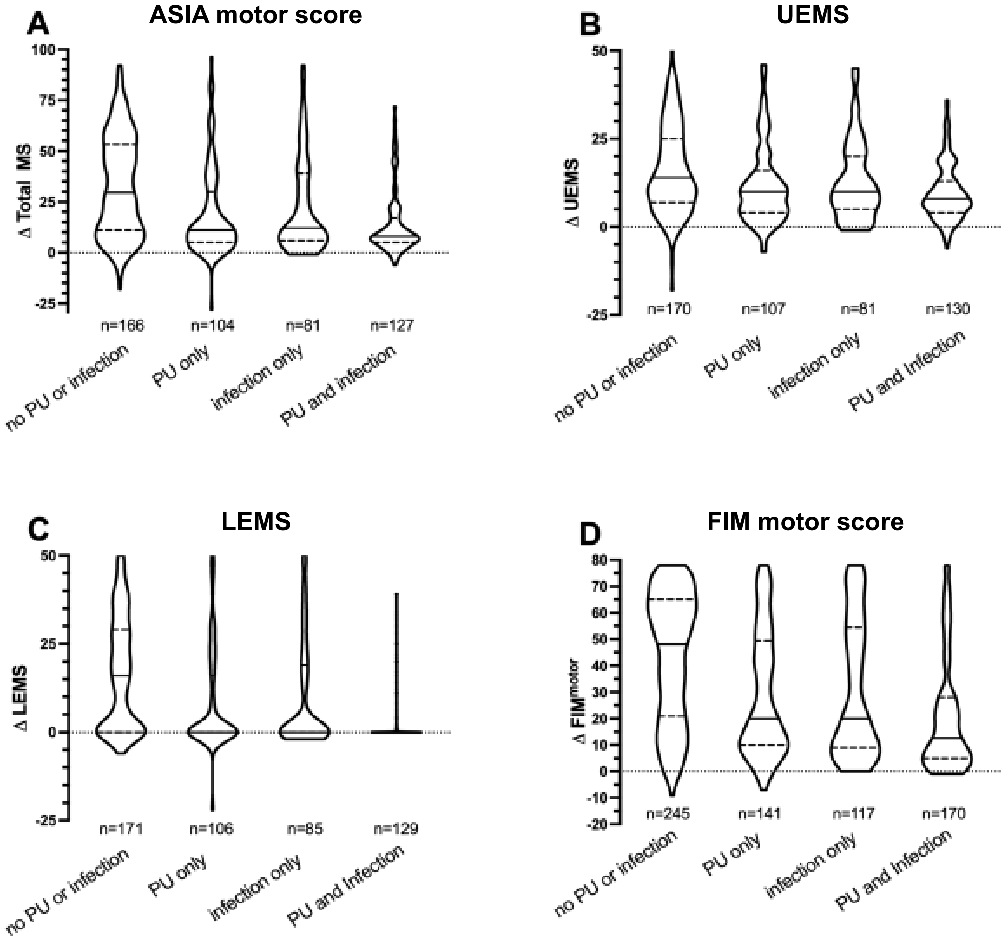

eFigure 8: Regarding the outcome modifying factor ‘infection’, more cases of pneumonia occurred during primary surgical and rehabilitative care in the group exposed to pressure ulcers [PU(+)] [n=340 of 586, (58,0%)] compared with the group unexposed to pressure ulcers [PU(-)] [n=224 of 693, (32,5%), n=3 missing]. Postoperative wound infections occurred rarely and were equally distributed in the PU(+) group [n=14 of 586, (2,4%)] and the PU(-) group [n=12 of 695, (1,7%), n=1 missing]. The violin plots indicate **(A)** ASIA motor score, **(B)** UEMS, **(C)** LEMS**,** and **(D)** FIM motor score differences from baseline to 1 year [13 (12 to 15) months after SCI, median (interquartile range)] in the total sample (AIS A, B, C). The effects of exposure to individual and combined outcome modifying factors ‘infection’ and ‘pressure ulcer’ was categorized and compared as follows: ‘no PU or infection’ vs. ‘PU only’ vs. ‘infection only’ vs. ‘PU and infection’. Groups were compared using the Kruskal-Wallis test with Bonferroni adjusted post-tests. **(A)** ASIA motor score p < 0.001; post-tests: ‘PU only’ vs. ‘no PU or infection’ p < 0.001, ‘infection only’ vs. ‘no PU or infection’ p = 0.002, ‘PU and infection vs. ‘no PU or infection’ p < 0.001, infection only’ vs. ‘PU only’ p = 1.0, PU only’ vs. ‘PU and infection’ p = 0.47, infection only’ vs. ‘PU and infection’ p = 0.11. **(B)** UEMS p < 0.001; post-tests: ‘PU only’ vs. ‘no PU or infection’ p = 0.005, ‘infection only’ vs. ‘no PU or infection’ p = 0.18, ‘PU and infection vs. ‘no PU or infection’ p < 0.001, infection only’ vs. ‘PU only’ p = 1.0, PU only’ vs. ‘PU and infection’ p =1.0, infection only’ vs. ‘PU and infection’ p = 0.41. **(C)**

© 2024 Kopp MA et al. *JAMA Network Open.*

LEMS p < 0.001; post-tests: ‘PU only’ vs. ‘no PU or infection’ p < 0.001, ‘infection only’ vs. ‘no PU or infection’ p < 0.001, ‘PU and infection vs. ‘no PU or infection’ p < 0.001, infection only’ vs. ‘PU only’ p = 1.0, PU only’ vs. ‘PU and infection’ p = 0.56, infection only’ vs. ‘PU and infection’ p = 0.087. **(D)** FIM motor score p < 0.001; post-tests: ‘PU only’ vs. ‘no PU or infection’ p < 0.001, ‘infection only’ vs. ‘no PU or infection’ p < 0.001, ‘PU and infection vs. ‘no PU or infection’ p < 0.001, infection only’ vs. ‘PU only’ p = 1.0, PU only’ vs. ‘PU and infection’ p = 0.004, infection only’ vs. ‘PU and infection’ p = 0.003. Acquisition of PU together with contracted infections demonstrate additive negative associations with ASIA motor score and FIM motor score recovery. Median/quartiles are indicated as solid/broken lines. Abbreviations: AIS = ASIA impairment scale, ASIA = American Spinal Injury Association, FIM = Functional Independence Measure, LEMS = Lower Extremity Motor Score, PU = pressure ulcer, UEMS = Upper Extremity Motor Score.

© 2024 Kopp MA et al. *JAMA Network Open.*

eFigure 9. Effect of hospital-acquired pressure ulcers on survival up to 10 years

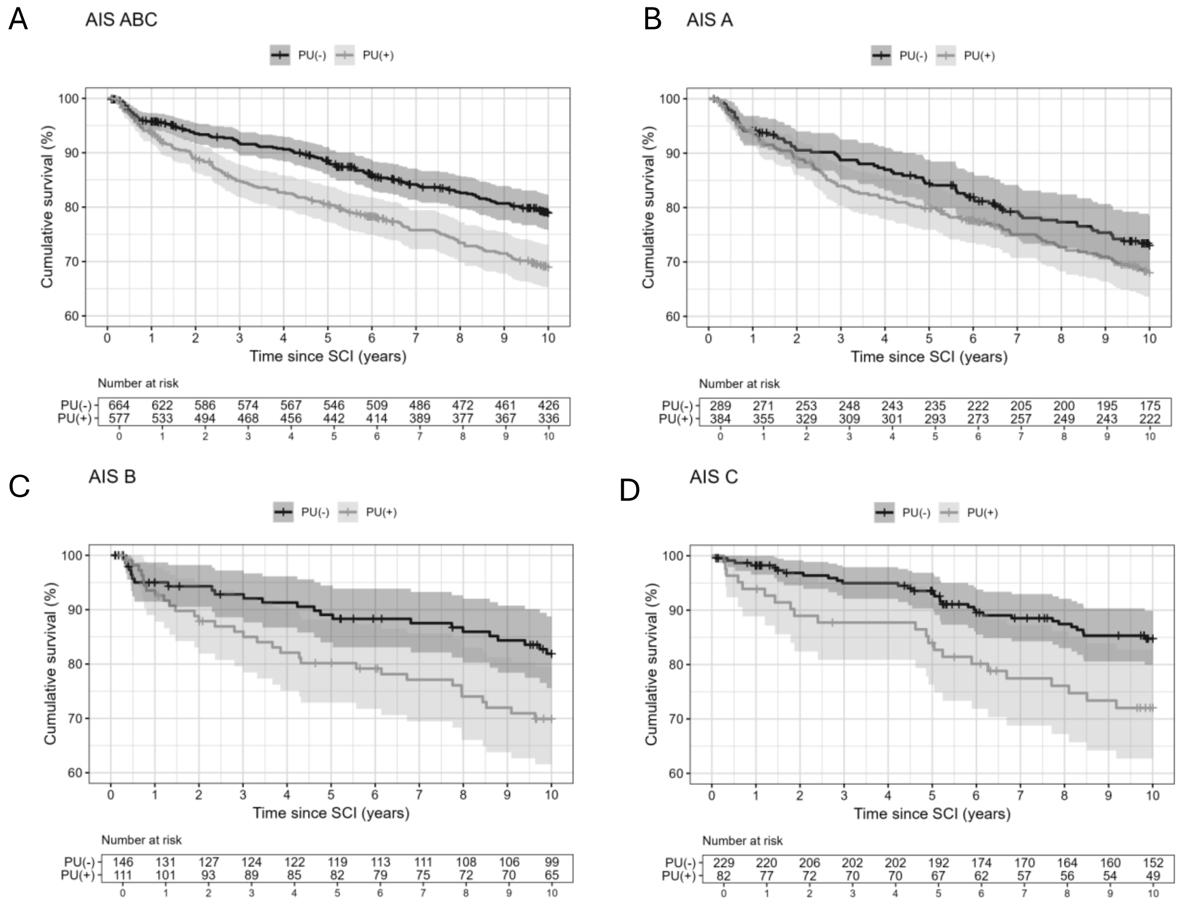

eFigure 9: Kaplan-Meier analysis comparing survival up to 10 years between unexposed patients without pressure ulcers [PU(-)] to patients exposed to pressure ulcers [PU(+)] in **(A)** the total sample (AIS A, B, C) and **(B-C)** the respective, individual AIS strata. Censored cases are indicated as vertical lines on the curves. Shadowed areas indicate the 95% confidence interval. Abbreviations: AIS = ASIA impairment scale, ASIA = American Spinal Injury Association, PU = pressure ulcer.

© 2024 Kopp MA et al. *JAMA Network Open.*

eTable 1. Variable definitions for neurological classification of spinal cord injury according to the SCIMS data dictionary 2000 to 2005

**American spinal injury association (ASIA) impairment scale (AIS)**
AIS A – Complete: No motor or sensory function is preserved in the sacral segments S4–S5.
AIS B – Incomplete: Sensory but not motor function is preserved below the neurological level and includes the sacral segments S4-5
AIS C – Incomplete: Motor function is preserved below the neurological level, and more than half of key muscles below the neurological level have a muscle grade less than 3.
AIS D – Incomplete: Motor function is preserved below the neurological level, and at least half of key muscles below the neurological level have a muscle grade of 3 or more.
AIS E – Normal: Motor and sensory function are normal.

**Neurological level of injury**
The neurological level of injury is the most caudal segment of the spinal cord with normal sensory and motor function on both sides of the body.

**ASIA motor scores**
The total ASIA motor score is composed of the grades of strength from 5 key muscles of the upper extremity (C5–T1) and 5 key muscles of the lower extremity (L2–S1) for either side of the body. The valid range of the total motor score is 100 points.
Muscle grading:
Grade 0 – Total paralysis
Grade 1 – Palpable or visible contraction
Grade 2 – Active movement, full range of motion, gravity eliminated.
Grade 3 – Active movement, full range of motion against gravity.
Grade 4 – Active movement, full range of motion against gravity and provides some resistance
Grade 5 – Active movement, full range of active motion against gravity and provides normal resistance.
The motor subscores (UEMS = upper extremity motor score) or (LEMS = lower extremity motor score) can be calculated as the sum of the grades of strength from 5 key muscles of the upper extremity (C5–T1) for either side of the body or 5 key muscles of the lower extremity (L2–S1) for either side of the body, respectively. The valid range of UEMS and LEMS is 50 points.

© 2024 Kopp MA et al. *JAMA Network Open.*

eTable 2. Grades of pressure ulcer as defined in the SCIMS data-dictionary 2000 to 2005

Grade	Definition
0	None and redness that does blanch to the touch.
1	Limited to the superficial epidermal and dermal layers. Include redness that does not blanch to the touch and redness that requires intervention.
2	Involving the epidermal and dermal layers and extending into the adipose tissue.
3	Extending through superficial structures and adipose tissue down to and including muscle.
4	Destruction of all soft tissue structures and communication with bone or joint structures.

© 2024 Kopp MA et al. *JAMA Network Open.*

eTable 3. Functional Independence Measure (FIM) motor score and subscores as defined in the SCIMS data-dictionary 2000 to 2005

Subscores	Subscore items	FIM^{motor} item scoring
**Self Care**	1. Eating<br>2. Grooming<br>3. Bathing<br>4. Dressing Upper Body<br>5. Dressing Lower Body<br>6. Toileting	*INDEPENDENT (no helper) - Another person is not required for the activity.*<br>7 Complete Independence - The subject safely performs all the tasks described as making up the activity within a reasonable amount of time, and does so without modification, assistive devices, or aids.<br>6 One or more of the following may be true: the activity requires an assistive device, the activity takes more than reasonable time, or the activity involves safety (risk) considerations.
**Sphincter Control**	1. Bladder Management<br>2. Bowel Management	*DEPENDENT (requires helper): Subject requires another person for either supervision or physical assistance in order for the activity.*<br>*Modified Dependence - The subject expends half (50%) or more of the effort.*
**Transfer**	1. Bed / Chair / Wheelchair<br>2. Toilet<br>3. Bathtub / Shower	5 Supervision or Setup – The subject requires no more help than standby, cueing or coaxing, without physical contact; alternately, the helper sets up needed items or applies orthoses or assistive/adaptive devices.<br>4 Minimal Contact Assistance – The subject requires no more help than touching and expends 75% or more of the effort.
**Locomotion**	1. Walking / Wheelchair<br>2. Stairs	3 Moderate Assistance – The subject requires more help than touching, or expends between 50 and 75% of the effort.<br>Complete Dependence - The subject expends less than half (less than 50%) of the effort. Maximal or total assistance is required.<br>2 Maximal Assistance – The subject expends between 25 and 49% of the effort.<br>1 Total Assistance – The subject expends less than 25% of the effort or subject cannot be rated due to physical or cognitive limitations and a helper performs the activity for the patient.

eTable3: The FIM motor score contains 13 motor items and 4 different FIM sub-scores, in detail: self-care, sphincter control, transfer and locomotion. For each item the score ranges from 1–7 points assessing the level of physical independence: ‘1’ means total assistance is necessary, then gradually differentiated up to ‘7’ (complete independence). The total FIM motor score ranges from 13–91 points.

© 2024 Kopp MA et al. *JAMA Network Open.*

eTable 4. Comparison of sociodemographic baseline characteristics of screened, included, and excluded patients

	Screened	Included	Excluded
Patients, No (percent)	3,654 (100)	1,282 (35.1)	2,372 (64.9)
Age, years; mean (95% CI)	37.8 (37.3 to 38.4)	38.0 (37.1 to 38.8)	37.8 (37.1 to 38.5)
Sex, male; percent (95% CI)	79.7 (78.4 to 81.0)	80.2 (78.0 to 82.4)	79.4 (77.8 to 81.0)
Ethnic group, White; percent (95% CI)	66.7 (65.2 to 68.2)	65.1 (62.5 to 67.7)	67.6 (65.7 to 69.5)
Native language, English; percent (95% CI)	94.0 (93.1 to 94.9)	93.8 (92.3 to 95.3)	94.1 (92.9 to 95.3)
Education, highschool or higher; percent (95% CI)	69.2 (67.5 to 70.9)	71.6 (68.9 to 74.3)	67.7 (65.5 to 69.9)
Occupational status, working/studying; percent (95% CI)	70.9 (69.3 to 72.5)	70.9 (68.3 to 73.5)	71.0 (68.9 to 73.1)
Marital status, married; percent (95% CI)	34.0 (32.3 to 35.7)	36.2 (33.5 to 38.9)	32.6 (30.5 to 34.7)

eTable 4: Explorative comparison of included (analyzed) cases with cases not analyzed did not reveal substantial differences in the structure of sociodemographic baseline characteristics between these groups. Continuous variables are reported as mean with 95% CI and categorical variables as relative frequency with 95% CI.

© 2024 Kopp MA et al. *JAMA Network Open.*

eTable 5. Comparison of sociodemographic and baseline characteristics of complete cases with patients lost to follow-up

	Total sample at baseline	ASIA motor score at 1 year		FIM motor score at 1 year	
Patients, No, (Percent)	1,282 (100)	Complete cases 478 (37.3)	Lost to follow-up 804 (62.7)	Complete cases 698 (54.5)	Lost to follow-up 584 (45.5)
Age, years; mean ± SD (95% CI)	38.0 (37.1 to 38.8)	36.0 (34.7 to 37.3)	39.1 (38.0 to 40.3)	36.9 (35.8 to 38.0)	39.2 (37.9 to 40.6)
Sex, male; percent (95% CI)	80.2 (78.0 to 82.4)	78.7 (75.0 to 82.4)	81.1 (78.4 to 83.8)	78.8 (75.8 to 81.8)	81.8 (78.7 to 84.9)
Ethnic group, White; percent (95% CI)	65.1 (62.5 to 67.7)	64.2 (59.9 to 68.5)	65.5 (62.2 to 68.8)	68.5 (65.1 to 71.9)	61.0 (57.0 to 65.0)
Native language, English; percent (95% CI)	93.8 (92.3 to 95.3)	92.2 (89.6 to 94.8)	95.0 (93.2 to 96.8)	93.1 (91.2 to 95.0)	95.1 (92.8 to 97.4)
Education, high school or higher; percent (95% CI)	71.6 (68.9 to 74.3)	70.3 (66.0 to 74.6)	72.5 (69.1 to 75.9)	73.0 (69.6 to 76.4)	69.4 (65.0 to 73.8)
Occupational status, working/studying; percent (95% CI)	70.9 (68.3 to 73.5)	77.3 (73.3 to 81.3)	66.9 (63.4 to 70.4)	75.5 (72.2 to 78.8)	64.1 (59.7 to 68.5)
Marital status, married; percent (95% CI)	36.2 (33.5 to 38.9)	34.7 (30.3 to 39.7)	37.2 (33.6 to 40.8)	36.5 (32.9 to 40.1)	35.8 (31.5 to 40.1)
Pressue ulcer status, PU(+) percent (95% CI)	45.7 (43.0 to 48.4)	48.3 (43.8 to 52,8)	44.2 (40.8 to 47.6)	45.0 (41.3 to 48.7)	46.6 (42.6 to 50.6)
AIS, A; percent (95% CI)	54.9 (52.2 to 57.6)	56.3 (51.9 to 60.7)	54.1 (50.7 to 57.5)	52.4 (48.7 to 56.1)	57.9 (53.9 to 61.9)
AIS, B; percent (95% CI)	20.4 (18.2 to 22.6)	20.9 (17.3 to 24.5)	20.0 (17.2 to 22.8)	21.1 (18.1 to 24.1)	19.5 (16.3 to 22.7)
AIS, C; percent (95% CI)	24.7 (22.3 to 27.1)	22.8 (19.0 to 26.6)	25.9 (22.9 to 28.9)	26.5 (23.2 to 29.8)	22.6 (19.2 to 26.0)
NLI, C1-C4, percent (95% CI)	51.8 (49.1 to 54.5)	47.5 (43.0 to 52.0)	54.4 (51.0 to 57.8)	49.9 (46.2 to 53.6)	54.1 (50.1 to 58.1)
ASIA motor score baseline; mean (95% CI)	19.7 (18.7 to 20.8)	19.7 (18.2 to 21.3)	19.8 (18.4 to 21.1)	20.2 (18.9 to 21.6)	19.2 (17.7 to 20.7)
FIM motor score baseline; mean (95% CI)	17.2 (16.8 to 17.7)	17.2 (16.5 to 17.9)	17.2 (16.6 to 17.8)	17.4 (16.8 to 18.1)	16.9 (16.3 to 17.6)

eTable 5: An analysis aiming to obtain indications for a possible attrition bias was performed by comparing the baseline characteristics of cases with available to those with missing ASIA motor score or FIM motor score follow-up data at 1 year. Based on 1,282 patients included, 478 (37.3%) records for the ASIA motor score and 698 (54.5%) for the FIM motor score were available at 1 year. Cases lost to follow-up for both outcomes were moderately older, less frequently employed/studying, and had a slightly higher rate of C1-C4 neurological level. For the FIM motor score, a discrepancy existed also for ethnic group with fewer White patients being lost to follow-up. The frequency of the exposure variable 'pressure ulcer' and major outcome relevant predictors such as the AIS, ASIA motor score and FIM motor score at baseline were not different between cases with complete and missing follow-up data. Continuous variables are reported as mean with 95% CI and categorical variables as relative frequency with 95% CI. Abbreviations: AIS = ASIA impairment scale, ASIA = American Spinal Injury Association, FIM = Functional Independence Measure motor items, NLI = Neurological level of injury, PU = pressure ulcer.

© 2024 Kopp MA et al. *JAMA Network Open.*

eTable 6. Association of hospital-acquired pressure ulcers with ASIA motor score recovery after Multiple Imputation

	Discharge (median, [interquartile range] 81 [56 to 117] days after SCI)			1 year (median, [interquartile range] 13 [12 to 15] months after SCI)		PU x Time	
**Model**	**Patients, No.**	**Exposed [PU(+)] vs unexposed [PU(-)] difference (95% CI)**	**p-value**	**Exposed [PU(+)] vs unexposed [PU(-)] difference (95% CI)**	**p-value**	**Change in difference [PU(+) - PU(-)] from discharge to 1 y (95% CI)**	**p-value**
1	1282	-6.5 (-9.0 to -4.1)	<0.001	-10.5 (-13.9 to -7.2)	<0.001	-4.0 (-6.9 to -1.1)	0.01
2	1282	-6.1 (-8.6 to -3.7)	<0.001	-10.1 (-13.4 to -6.7)	<0.001	-3.9 (-6.8 to -1.0)	0.01
3	1282	-3.3 (-5.4 to -1.2)	0.002	-6.7 (-9.9 to -3.4)	<0.001	-3.4 (-6.4 to -0.4)	0.03
4A	704	-1.6 (-3.8 to 0.7)	0.17	-3.0 (-6.7 to 0.6)	0.10	-1.5 (-4.9 to 2.0)	0.39
4B	261	-7.2 (-13.4 to -1.1)	0.02	-7.8 (-15.0 to -0.6)	0.03	-0.6 (-6.3 to 5.2)	0.84
4C	317	-6.4 (-11.4 to -1.4)	0.01	-18.4 (-25.1 to -11.6)	<0.001	-11.9 (-18.2 to -5.7)	<0.001

eTable 6: Linear mixed regression models of the ASIA motor score change from baseline after Multiple Imputation of missing data. The table indicates the differences in motor score recovery between the group exposed to PUs [PU(+)] and the group unexposed to pressure ulcers [PU(-)] at discharge from inpatient rehabilitation and 1 year follow-up expressed as difference of estimated marginal means with 95% CI and p-value. In addition, the change of the estimated differences between the groups from discharge from inpatient rehabilitation to 1 year is reported. PU-group interaction with time is reported. The models were calculated adjusted for the motor score baseline **(model 1)**, additionally adjusted for sociodemographic variables (age, sex, educational status, language status, occupational status) and NLI **(model 2)**, and subsequently further adjusted for the AIS **(model 3)**. In a sensitivity analysis model 2 was stratified for the AIS **(model 4A, 4B, 4C).** Center is considered as a random effect for all models, except for model 4B due to convergence problems. No. describes number of patients with complete information on selected model variables after multiple imputation (30 complete data sets). Goodness of fit (mean of m=30 models) expressed as conditional R^2 (R^2c for fixed and random effects) and marginal R^2 (R^2m for fixed effects): model 1: $R^2c = 0.89$, $R^2m = 0.50$; model 2: $R^2c = 0.89$, $R^2m = 0.52$; model 3: $R^2c = 0.89$, $R^2m = 0.64$; model 4A: $R^2c = 0.82$, $R^2m = 0.47$; model 4B: $R^2c = 0.84$, $R^2m = 0.29$; model 4C: $R^2c = 0.80$, $R^2m = 0.40$. Abbreviations: 95% CI = 95% Confidence Interval, AIS = ASIA impairment scale, ASIA = American Spinal Injury Association, NLI = Neurological level of injury, PU = pressure ulcer.

© 2024 Kopp MA et al. *JAMA Network Open.*

eTable 7. Association of hospital-acquired pressure ulcers with UEMS recovery

	Discharge (median, [interquartile range] 81 [56 to 117] days after SCI)			1 year (median, [interquartile range] 13 [12 to 15] months after SCI)			PU x Time	
**Model**	**Patients, No.**	**Exposed [PU(+)] vs unexposed [PU(-)] difference (95% CI)**	**p-value**	**Patients, No.**	**Exposed [PU(+)] vs unexposed [PU(-)] difference (95% CI)**	**p-value**	**Change in difference [PU(+) - PU(-)] from discharge to 1 y (95% CI)**	**p-value**
1	1157	-2.8 (-3.9 to -1.6)	<0.001	478	-5.6 (-7.0 to -4.2)	<0.001	-2.9 (-4.0 to -1.7)	<0.001
2	803	-3.3 (-4 6 to -2.0)	<0.001	381	-6.2 (-7.9 to -4.6)	<0.001	-2.9 (-4.3 to -1.6)	<0.001
3	803	-1.4 (-2.7 to -0.1)	0.03	381	-3.7 (-5.3 to -2.1)	<0.001	-2.3 (-3.7 to -0.9)	0.001
4A	451	-0.7 (-2.3 to 0.8)	0.34	221	-2.2 (-4.1 to -0.4)	0.02	-1.5 (-3.0 to 0.0)	0.06
4B	151	-3.0 (-5.9 to -0.1)	0.05	70	-5.8 (-9.6 to -2.0)	0.003	-2.8 (-6.0 to 0.4)	0.10
4C	201	-2.2 (-5.1 to 0.8)	0.16	90	-6.3 (-10.5 to -2.2)	0.003	-4.2 (-8.0 to -0.4)	0.04

eTable 7: Linear mixed regression models of the UEMS change from baseline. The table indicates the differences in UEMS recovery between the group exposed to PUs [PU(+)] and the group unexposed to pressure ulcers [PU(-)] at discharge from inpatient rehabilitation [81 (56 to 117) days after SCI, median, (interquartile range)] and 1 year follow-up [13 (12 to 15) months after SCI, median (interquartile range)] expressed as difference of estimated marginal means with 95% CI and p-value. In addition, the change of the estimated differences between the groups from discharge from inpatient rehabilitation to 1 year is reported. PU-group interaction with time is reported. The models were calculated adjusted for the motor score baseline **(model 1)**, additionally adjusted for sociodemographic variables (age, sex, educational status, language status, occupational status) and NLI **(model 2)**, and subsequently further adjusted for the AIS **(model 3)**. In a sensitivity analysis model 2 was stratified for the AIS **(model 4A, 4B, 4C).** Center is considered as a random effect for all models, except for model 4B due to convergence problems. No. describes number of patients with complete information on selected model variables at discharge or 1 year. Goodness of fit expressed as conditional R^2 (R^2c for fixed and random effects) and marginal R^2 (R^2m for fixed effects): model 1: R^2c = 0.91, R^2m = 0.62; model 2: R^2c = 0.86, R^2m = 0.53; model 3: R^2c = 0.86, R^2m = 0.65; model 4A: R^2c = 0.93, R^2m = 0.73; model 4B: R^2c = 0.87, R^2m = 0.57; model 4C: R^2c = 0.77, R^2m = 0.41. Abbreviations: 95% CI = 95% Confidence Interval, AIS = ASIA impairment scale, ASIA = American Spinal Injury Association, NLI = Neurological level of injury, PU = pressure ulcer, UEMS = Upper Extremity Motor Score.

© 2024 Kopp MA et al. *JAMA Network Open.*

eTable 8. Association of hospital-acquired pressure ulcers with LEMS recovery

	Discharge (median, [interquartile range] 81 [56 to 117] days after SCI)			1 year (median, [interquartile range] 13 [12 to 15] months after SCI)			PU x Time	
**Model**	**Patients, No.**	**Exposed [PU(+)] vs unexposed [PU(-)] difference (95% CI)**	**p-value**	**Patients, No.**	**Exposed [PU(+)] vs unexposed [PU(-)] difference (95% CI)**	**p-value**	**Change in difference [PU(+) - PU(-)] from discharge to 1 y (95% CI)**	**p-value**
1	1157	-4.0 (-5.5 to -2.6)	<0.001	478	-7.6 (-9.5 to -5.7)	<0.001	-3.6 (-5.2 to -1.9)	<0.001
2	803	-5.0 (-6.8 to -3.2)	<0.001	381	-8.5 (-10.7 to -6.3)	<0.001	-3.5 (-5.4 to -1.6)	<0.001
3	803	-2.9 (-4.5 to -1.3)	<0.001	381	-5.7 (-7.7 to -3.7)	<0.001	-2.8 (-4.7 to -0.9)	0.004
4A	451	-1.1 (-2.7 to 0.5)	0.17	221	-3.5 (-5.5 to -1.5)	<0.001	-2.4 (-4.3 to -0.5)	0.01
4B	151	-6.7 (-11.6 to -1.8)	0.01	70	-7.5 (-13.7 to -1.3)	0.02	-0.8 (-5.9 to 4.3)	0.77
4C	201	-2.8 (-6.8 to 1.2)	0.16	90	-8.5 (-14.2 to -2.8)	0.003	-5.7 (-11.2 to -0.1)	0.05

eTable 8: Linear mixed regression models of the LEMS change from baseline. The table indicates the differences in LEMS recovery between the group exposed to PUs [PU(+)] and the group unexposed to pressure ulcers [PU(-)] at discharge from inpatient rehabilitation [81 (56 to 117) days after SCI, median, (interquartile range)] and 1 year follow-up [13 (12 to 15) months after SCI, median (interquartile range)] expressed as difference of estimated marginal means with 95% CI and p-value. In addition, the change of the estimated differences between the groups from discharge from inpatient rehabilitation to 1 year is reported. PU-group interaction with time is reported. The models were calculated adjusted for the motor score baseline **(model 1)**, additionally adjusted for sociodemographic variables (age, sex, educational status, language status, occupational status) and NLI **(model 2)**, and subsequently further adjusted for the AIS **(model 3)**. In a sensitivity analysis model 2 was stratified for the AIS **(model 4A, 4B, 4C).** Center is considered as a random effect for all models, except for model 4B due to convergence problems. No. describes number of patients with complete information on selected model variables at discharge or 1 year. Goodness of fit expressed as conditional R^2 (R^2c for fixed and random effects) and marginal R^2 (R^2m for fixed effects): model 1: $R^2c = 0.86$, $R^2m = 0.52$; model 2: $R^2c = 0.86$, $R^2m = 0.53$; model 3: $R^2c = 0.86$, $R^2m = 0.65$; model 4A: $R^2c = 0.65$, $R^2m = 0.14$; model 4B: $R^2c = 0.78$, $R^2m = 0.21$; model 4C: $R^2c = 0.69$, $R^2m = 0.42$. Abbreviations: 95% CI = 95% Confidence Interval, AIS = ASIA impairment scale, ASIA = American Spinal Injury Association, NLI = Neurological level of injury, LEMS = Lower Extremity Motor Score, PU = pressure ulcer.

© 2024 Kopp MA et al. *JAMA Network Open.*

eTable 9. Improvement in FIM subscores and total FIM motor score from baseline to 1 year follow-up

Discharge (median, [interquartile range] 81 [56 to 117]

	AIS ABC				AIS A			
	Patients, No.	Unexposed [PU(-)]	Exposed [PU(+)]	p-value	Patients, No.	Unexposed [PU(-)]	Exposed [PU(+)]	p-value
Self-Care	1,170	12.0 (5.0 to 19.0)	7.0 (0.0 to 13.0)	<0.001	630	7.0 (1.0 to 13.8)	6.0 (0.0 to 12.0)	0.014
Sphincter Control	1,174	0.0 (0.0 to 8.0)	0.0 (0.0 to 1.0)	<0.001	630	0.0 (0.0 to 1.0)	0.0 (0.0 to 0.0)	0.098
Transfers	1,174	6.0 (1.0 to 11.0)	1.0 (0.0 to 5.0)	<0.001	635	2.0 (0.0 to 5.0)	1.0 (0.0 to 3.0)	0.037
Locomotion	1.173	5.0 (2.0 to 6.0)	4.0 (1.0 to 5.0)	<0.001	634	5.0 (2.0 to 5.0)	4.0 (0.0 to 5.0)	0.004
FIM motor score	1,157	23.0 (9.0 to 40.0)	11.0 (4.0 to 23.3)	<0.001	626	12.0 (5.0 to 22.8)	9.5 (3.0 to 19.0)	0.001

	AIS B				AIS C			
	Patients, No.	Unexposed [PU(-)]	Exposed [PU(+)]	p-value	Patients, No.	Unexposed [PU(-)]	Exposed [PU(+)]	p-value
Self-Care	241	13.0 (7.0 to 20.0)	9.0 (2.5 to 15.5)	0.001	299	16.0 (9.0 to 24.0)	9.0 (3.8 to 18.0)	<0.001
Sphincter Control	244	2.0 (0.0 to 8.0)	0.0 (0.0 to 2.0)	0.001	300	6.0 (1.0 to 10.0)	0.0 (0.0 to 8.0)	<0.001
Transfers	242	6.0 (1.0 to 11.0)	2.0 (0.0 to 7.3)	<0.001	297	9.0 (6.0 to 13.0)	4.0 (0.0 to 12.3)	<0.001
Locomotion	242	5.0 (2.0 to 6.0)	5.0 (1.0 to 5.0)	0.111	297	6.0 (3.0 to 8.0)	5.0 (2.0 to 6.0)	0.001
FIM motor score	237	27.5 (11.8 to 41.0)	13.0 (5.0 to 32.0)	<0.001	294	36.0 (24.0 to 51.5)	20.0 (7.0 to 44.0)	<0.001

1 year (median, [interquartile range] 13 [12 to 15] months after SCI)

	AIS ABC				AIS A			
	Patients, No.	Unexposed [PU(-)]	Exposed [PU(+)]	p-value	Patients, No.	Unexposed [PU(-)]	Exposed [PU(+)]	p-value
Self-Care	697	18.0 (7.0 to 29.0)	9.0 (2.0 to 18.8)	<0.001	369	9.0 (4.0 to 20.0)	6.0 (0.0 to 15.0)	0.002
Sphincter Control	698	6.0 (0.0 to 11.0)	0.0 (0.0 to 6.3)	<0.001	368	0.0 (0.0 to 7.0)	0.0 (0.0 to 4.0)	0.087
Transfers	680	11.0 (1.0 to 17.0)	2.0 (0.0 to 11.0)	<0.001	358	2.0 (0.0 to 11.3)	0.0 (0.0 to 6.0)	0.016
Locomotion	684	5.0 (5.0 to 10.0)	5.0 (4.0 to 5.0)	<0.001	362	5.0 (4.0 to 5.5)	5.0 (2.0 to 5.0)	0.023
FIM motor score	674	41.0 (14.0 to 62.0)	17.0 (7.0 to 41.0)	<0.001	356	16.5 (9.0 to 41.8)	12.0 (5.0 to 27.0)	0.014

	AIS B				AIS C			
	Patients, No.	Unexposed [PU(-)]	Exposed [PU(+)]	p-value	Patients, No.	Unexposed [PU(-)]	Exposed [PU(+)]	p-value
Self-Care	146	20.0 (12.0 to 29.0)	13.0 (4.0 to 25.0)	0.013	182	27.0 (17.0 to 33.0)	20.0 (6.5 to 32.5)	0.056
Sphincter Control	147	7.0 (0.0 to 11.0)	2.0 (0.0 to 10.0)	0.082	183	10.0 (6.0 to 12.0)	5.0 (0.0 to 11.0)	0.001
Transfers	141	14.0 (2.8 to 17.3)	6.0 (0.0 to 15.0)	0.011	181	15.0 (10.0 to 18.0)	11.0 (1.0 to 18.0)	0.020
Locomotion	143	5.0 (5.0 to 10.0)	5.0 (4.0 to 7.0)	0.086	179	9.0 (5.0 to 11.0)	6.0 (5.0 to 10.0)	0.046
FIM motor score	140	47.5 (21.5 to 65.0)	21.5 (9.8 to 57.8)	0.014	178	60.0 (41.0 to 69.5)	42.0 (12.0 to 69.0)	0.017

eTable 9: The score change is reported as median and Interquartile range. The unexposed group without pressure ulcers [PU(-)] and the exposed group with pressure ulcers [PU(-)] were compared using the Mann-Whitney-U-Test. Abbreviations: AIS = ASIA impairment scale, ASIA = American Spinal Injury Association, FIM = Functional Independence Measure, PU = pressure ulcer.

.

© 2024 Kopp MA et al. *JAMA Network Open.*

eReferences

1. Failli V, Kopp MA, Gericke C, et al. Functional neurological recovery after spinal cord injury is impaired in patients with infections. *Brain.* 2012;135(Pt 11):3238-3250.
2. Kopp MA, Watzlawick R, Martus P, et al. Long-term functional outcome in patients with acquired infections after acute spinal cord injury. *Neurology.* 2017;88(9):892-900.
3. van Buuren S, Groothuis-Oudshoorn K. mice: Multivariate Imputation by Chained Equations in R. *Journal of Statistical Software.* 2011;45(3):1-67.
4. White IR, Royston P. Imputing missing covariate values for the Cox model. *Stat Med.* 2009;28(15):1982-1998.
5. Krishnan S, Karg PE, Boninger ML, et al. Early Detection of Pressure Ulcer Development Following Traumatic Spinal Cord Injury Using Inflammatory Mediators. *Arch Phys Med Rehabil.* 2016;97(10):1656-1662.
6. Jogia T, Lubstorf T, Jacobson E, et al. Prognostic value of early leukocyte fluctuations for recovery from traumatic spinal cord injury. *Clin Transl Med.* 2021;11(1):e272.
7. Brommer B, Engel O, Kopp MA, et al. Spinal cord injury-induced immune deficiency syndrome enhances infection susceptibility dependent on lesion level. *Brain.* 2016;139(Pt 3):692-707.
8. Fawcett JW, Curt A, Steeves JD, et al. Guidelines for the conduct of clinical trials for spinal cord injury as developed by the ICCP panel: spontaneous recovery after spinal cord injury and statistical power needed for therapeutic clinical trials. *Spinal Cord.* 2007;45(3):190-205.
9. Kopp MA, Lubstorf T, Blex C, et al. Association of age with the timing of acute spine surgery-effects on neurological outcome after traumatic spinal cord injury. *Eur Spine J.* 2022;31(1):56-69.
10. Tennant PWG, Murray EJ, Arnold KF, et al. Use of directed acyclic graphs (DAGs) to identify confounders in applied health research: review and recommendations. *Int J Epidemiol.* 2021;50(2):620-632.
11. Kirshblum SC, Waring W, Biering-Sorensen F, et al. Reference for the 2011 revision of the International Standards for Neurological Classification of Spinal Cord Injury. *J Spinal Cord Med.* 2011;34(6):547-554.
12. Furlan JC, Fehlings MG, Tator CH, Davis AM. Motor and sensory assessment of patients in clinical trials for pharmacological therapy of acute spinal cord injury: psychometric properties of the ASIA Standards. *J Neurotrauma.* 2008;25(11):1273-1301.
13. Marino RJ, Graves DE. Metric properties of the ASIA motor score: subscales improve correlation with functional activities. *Arch Phys Med Rehabil.* 2004;85(11):1804-1810.
14. Cragg JJ, Tong B, Jutzeler CR, et al. A Longitudinal Study of the Neurologic Safety of Acute Baclofen Use After Spinal Cord Injury. *Neurotherapeutics.* 2019;16(3):858-867.
15. Geisler FH, Coleman WP, Grieco G, Poonian D, Sygen Study G. The Sygen multicenter acute spinal cord injury study. *Spine (Phila Pa 1976).* 2001;26(24 Suppl):S87-98.
16. Anderson KD, Acuff ME, Arp BG, et al. United States (US) multi-center study to assess the validity and reliability of the Spinal Cord Independence Measure (SCIM III). *Spinal Cord.* 2011;49(8):880-885.

© 2024 Kopp MA et al. *JAMA Network Open.*
