# Supplementary material for: Hospital-Acquired Pressure Ulcers and Long-Term Motor Score Recovery in Patients With Acute Cervical Spinal Cord Injury
Source: JAMA Netw Open. 2024 Dec 6;7(12):e2444983. doi: 10.1001/jamanetworkopen.2024.44983 (PMC11624580; doi:10.1001/jamanetworkopen.2024.44983)
Supplement: Supplement 2. — Data Sharing Statement [file jamanetwopen-e2444983-s002.pdf]

Data Sharing Statement

Kopp. Hospital-Acquired Pressure Ulcers and Long-Term Motor Score Recovery in Patients With Acute Cervical Spinal Cord Injury. *JAMA Netw Open*. Published December 06, 2024. doi:10.1001/jamanetworkopen.2024.44983

Data
Data available: Yes
Data types: Deidentified participant data, Data dictionary
How to access data: https://www.nscisc.uab.edu/Research/NSCISC_DatabasePublicUse
When available: With publication

Supporting Documents
Document types: Statistical/analytic code
How to access documents: marcel.kopp@charite.de
When available: With publication

Additional Information
Who can access the data: Researchers whose proposed use of the data has been approved
Types of analyses: For non-commercial research
Mechanisms of data availability: With investigator support via online data request form
